# Lysophosphatidylcholine acyltransferase 2-mediated lipid droplet production supports colorectal cancer chemoresistance

**DOI:** 10.1038/s41467-017-02732-5

**Published:** 2018-01-22

**Authors:** Alexia Karen Cotte, Virginie Aires, Maxime Fredon, Emeric Limagne, Valentin Derangère, Marion Thibaudin, Etienne Humblin, Alessandra Scagliarini, Jean-Paul Pais de Barros, Patrick Hillon, François Ghiringhelli, Dominique Delmas

**Affiliations:** 1 University of Bourgogne-Franche Comté, F-21000 Dijon, France; 2 INSERM U1231, Lipids, Nutrition, Cancer, F-21000 Dijon, France; 3 Research Team CADIR, Cancer and Adaptative Immune Response, F-21000 Dijon, France; 4 Transfer platform in cancer biology, Department of biology and tumour pathology, Georges François Leclerc Centre, F-21000 Dijon, France; 5 Lipidomic Platform, F-21000 Dijon, France; 6 Department of Hepatogastroenterology, University Hospital, F-21000 Dijon, France; 7 Department of medical Oncology, Georges François Leclerc Centre, F-21000 Dijon, France

## Abstract

Lipid droplet (LD) accumulation is a now well-recognised hallmark of cancer. However, the significance of LD accumulation in colorectal cancer (CRC) biology is incompletely understood under chemotherapeutic conditions. Since drug resistance is a major obstacle to treatment success, we sought to determine the contribution of LD accumulation to chemotherapy resistance in CRC. Here we show that LD content of CRC cells positively correlates with the expression of lysophosphatidylcholine acyltransferase 2 (LPCAT2), an LD-localised enzyme supporting phosphatidylcholine synthesis. We also demonstrate that LD accumulation drives cell-death resistance to 5-fluorouracil and oxaliplatin treatments both in vitro and in vivo. Mechanistically, LD accumulation impairs caspase cascade activation and ER stress responses. Notably, droplet accumulation is associated with a reduction in immunogenic cell death and CD8^+^ T cell infiltration in mouse tumour grafts and metastatic tumours of CRC patients. Collectively our findings highlight LPCAT2-mediated LD accumulation as a druggable mechanism to restore CRC cell sensitivity.

## Abstract (web summary)

Lipid droplets (LD) accumulation correlates with colorectal cancer (CRC) relapse. Here the authors show that chemotherapy induces LD synthesis via acyltransferase LPCAT2 which, in turn, promotes chemoresistance via LD accumulation both in vitro and in vivo by blocking chemotherapy-induced ER stress.

## Introduction

Metabolic reprogramming is a common feature of cancer progression and metastasis^1^. Besides the Warburg effect, tumour cells also undergo lipid remodelling mostly characterised by aberrant de novo lipogenesis, cholesterogenesis due to oncogenic-driven lipogenic enzyme overexpression (e.g., fatty-acid synthase (FASN), 3-hydroxy-3-methylglutaryl-CoA reductase (HMGCR)). This bulk of newly synthesised lipids serves for membrane biogenesis and synthesis of essential lipid-derived second messengers (e.g., phosphatidic acid, phosphoinositides, eicosanoids, including prostaglandin E2 (PGE2)) to maintain cancer cell proliferation and survival^1–3^.

Aside from a boost in de novo lipid biosynthesis, lipid droplet (LD) accumulation has been observed in increasing numbers of cancer cell lines and neoplastic tissues^4–7^. This LD accumulation in non-adipocytic tissues has, in very recent years, emerged as a new hallmark of cancer. However, the relative contribution of LD accumulation in many aspects of cancer biology remains incompletely understood. LDs are dynamic organelles that either store excess lipids or fuel cells with essential lipids to sustain lipid homeostasis depending on energy requirements. They are composed of a neutral lipid core (triglycerides (TGs) and sterol-esters) surrounded by a phospholipid monolayer mainly composed of phosphatidylcholine (PC) and a broad range of proteins mainly involved in lipid metabolism^8^. The hydrophobic core of the LD is produced by the main TG pathway called the glycerol-phosphate pathway, which terminates in both diacylglycerol O-acyltransferase enzymes DGAT1 and DGAT2, located in the endoplasmic reticulum (ER)^9^. Mature LDs continue growing with ER interactions and production of PC by the enzymes of the Kennedy pathway, especially phosphocholine cytidylyltransferase alpha (CCTα) directly located in the LD monolayer^10^. The remodelling of PC species occurs with the re-acylation of lysophosphatidylcholine (LPC) by the enzymes of the Lands cycle: specifically, lysophosphatidylcholine acyltransferase LPCAT1 and LPCAT2 isoforms participating in LD expansion and stability^11^. These organelles have been shown to promote proliferation^12^ or survival under nutrient stress^13,14^, to reduce intracellular lipotoxicity^15^. They are also involved in inflammatory processes by producing proinflammatory lipid mediators such as PGE_2_^16^. Although a role for LD accumulation in tumour cell chemoresistance mechanisms has been suggested in some studies, no direct evidence has been provided so far^17^. For instance, it has been recently shown by label-free Raman spectroscopy that LD accumulation is a characteristic of colorectal cancer (CRC) stem cells, suggesting a potential implication of LD biogenesis in CRC relapse and its potential use as a biomarker in this cancer^18^.

Herein, we sought to fill in the gaps in the literature and explore LD formation and function under chemotherapy conditions in CRC cell models. We show both in vitro and in vivo that the Lands cycle acyltransferase LPCAT2 plays a crucial role in CRC cell LD production. In addition, we show that LPCAT2 overexpression and LD overproduction confer CRC cell chemoresistance by blocking chemotherapy-induced ER stress, calreticulin (CRT) membrane translocation and subsequent immunogenic cell death (ICD).

## Results

### LD production in CRC cell lines is driven by LPCAT2

**Fig. 1:**
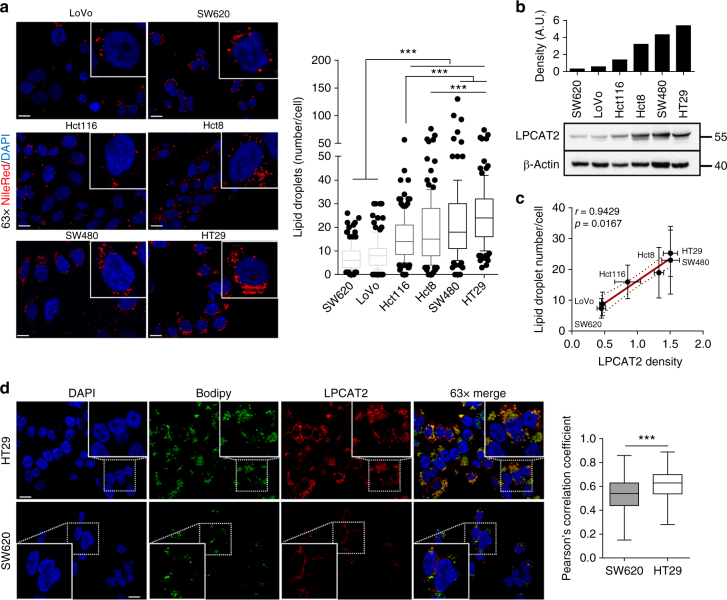
LD production correlates with LPCAT2 expression in CRC cell lines. **a** Basal LD content assessed by Nile red staining after 48 h of seeding. Left panel, representative confocal images of Nile red staining (×63 magnification, scale bar = 10 µm). Nuclei (blue), LD (red). Right panel, LD quantification was performed by counting red lipid bodies on merged pictures (300 cells per cell line) with Image J software (right). Whiskers denote 1^st^ and 99^th^ percentiles. *P* values were determined by a two-way ANOVA with Bonferroni correction. ***p < 0.001. **b** LPCAT2 basal expression. A representative blot from three independent experiments is shown. **c** A significant Spearman correlation coefficient (*p* = 0.0167) was found between basal LPCAT2 protein density and LD content from three independent experiments. **d** SW620 and HT29 cells were stained after 48 h of seeding with Bodipy 493/503 (green) and LPCAT2 antibody (red), and counterstained with DAPI (blue). The Pearson correlation coefficient was calculated between LPCAT2 and Bodipy fluorescences

We first evaluated and compared the basal LD content of six human colorectal cancer (CRC) cell lines (SW620, LoVo, Hct116, Hct8, SW480 and HT29) by intracellular neutral lipid staining with Nile red. Qualitative and quantitative analyses of the staining showed differential basal LD density, allowing the discrimination between tumour cells with low- and high-LD content **(**Fig. 1a**)**. Both phenotypes were further confirmed by transmission electron microscopy (TEM) analyses (Supplementary Fig. 1a) and quantification of cellular triglyceride (TG) levels (Supplementary Fig. 1b) in SW620 and HT29 cells. We next investigated whether the expression of key enzymes of PC biosynthetic pathways could account for the LD pattern observed. PC synthesis is achieved by two main routes: the Kennedy pathway supporting de novo PC production, and the Lands cycle involved in phospholipid remodelling through deacylation/re-acylation steps^9,10^. The key enzymes of the Kennedy pathway are as follows: choline kinase alpha (CKα), CTP:phosphocholine cytidylyltransferase (CCTα) and choline phosphotransferase 1 (CHPT1), CCTα being the rate-limiting enzyme. In the Lands cycle, LPCAT (isoforms 1,2,4) are the key enzymes connecting Lands and Kennedy pathways because they allow re-acylation of LPC at the *sn-*2 position to yield PC^9,10^. We found that both Kennedy and Lands cycle enzymes were differentially expressed across CRC cell lines (Supplementary Fig. 2a, b); nevertheless, only LPCAT2 expression was positively and significantly (Spearman’s correlation *p* value = 0.0167) correlated with basal LD content (Fig. 1b, c; Supplementary Fig. 2a, b). To further characterise the intimate correlation between LPCAT2 expression and LD production, we selected CRC cell lines with opposite phenotypes: SW620 cells, which have both the lowest basal LD content and LPCAT2 expression, which we refer to as low-LD CRC cells, and HT29 cells, which have both the largest LD content and strongest LPCAT2 expression, which we refer to as high-LD CRC cells.

**Fig. 2:**
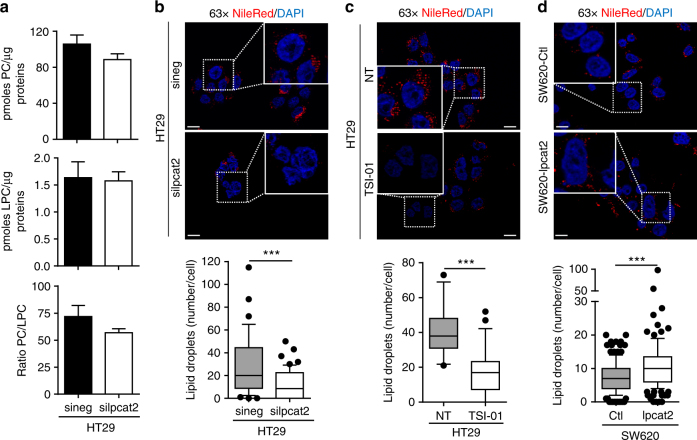
LD production depends on LPCAT2 in CRC cell lines. **a** HT29 cells’ PC and LPC levels assessed 72 h post-transfection with *Lpcat2* siRNA (silpcat2, 10 nM) or with scrambled siRNA (sineg, 10 nM). The data were normalised to the protein content of each sample and expressed as mean ± s.e.m. **b**–**d** LD staining with Nile red ( × 63 magnification, scale bar = 10 µm) in (**b**), HT29 cells 72 h post-transfection with silpcat2 or with sineg; (**c**), HT29 cells treated for 48 h with vehicle (DMSO) or selective LPCAT2 inhibitor, TSI-01 (10 µM); (**d**), SW620-Ctl vs. SW620 overexpressing LPCAT2 (SW620-lpcat2); number of LDs per cell and significance was evaluated by counting red lipid bodies on merged pictures (300 cells per condition) with Image J software. **a**–**d** The data presented are the combined results of three independent experiments. *P* values were determined by the Mann–Whitney *U* test. ***p < 0.001

It was shown that LPCAT1 and LPCAT2 enzymes, primarily located in the ER, can be recruited to the LD surface where they locally contribute to PC production^11,19^. Herein we show, with LPCAT2 fluorescent immunostaining and specific LD staining with Bodipy 493/503, that LPCAT2 co-localises with LD in high-LD CRC cells (HT29), whereas in low-LD CRC cells (SW620) LPCAT2 expression seemed more restricted to the ER compartment (Fig. 1d). HPLC-MS/MS analysis of PC and LPC species confirmed that, in agreement with increased LPCAT2 expression, PC metabolism was preponderant in high-LD CRC cells, as evidenced by the increase in some PC species (Supplementary Table 1). SiRNA-mediated downregulation of *LPCAT2* expression in HT29 cells was accompanied, accordingly, by a decrease in major PC species despite no significant impact on the total PC/LPC ratio (Fig. 2a; Supplementary Fig. 3a, b). Besides, *LPCAT2* silencing in high-LD CRC cells went along with a significant decrease in LD density (Fig. 2b), a phenotype that we also observed when the acyltransferase activity of LPCAT2 was pharmacologically and specifically blocked by TSI-01 (Fig. 2c). Conversely, stable LPCAT2 overexpression in SW620 cells (SW620-lpcat2) enhanced basal LD content as compared to mock infected control cells (pCMV6-empty vector, SW620-Ctl) (Fig. 2d; Supplementary Fig. 3d). Moreover, neither LPCAT2 inhibitor nor LPCAT2 overexpression significantly modulated TG levels (Supplementary Fig. 3c and e).

Altogether, these data demonstrate that LPCAT2 is essential for LD production in CRC cells.

### FOX chemotherapy triggers LPCAT2-dependent LD production

**Fig. 3:**
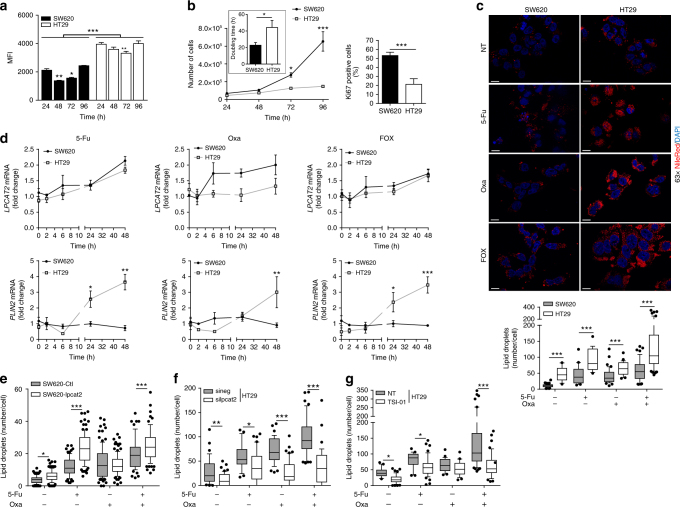
LPCAT2 supports 5-Fu and Oxa-induced LD production. **a** SW620 and HT29 cells were stained with Bodipy 493/503 and analysed by flow cytometry after 24, 48, 72 and 96 h of seeding. Bodipy 493/503 median of fluorescence intensity (MFI) was used to assess LD content. The multiple Student *t* test was used to compare cell lines at each time point and a two-way ANOVA with Bonferroni correction was used to compare MFI for each cell line at each time point (MFI at 24 h being used as control). ***p < 0.001. Error bars denote s.e.m. **b** SW620 *vs* HT29 cell growth curves (left panel). Doubling time was determined by the following equation: DT = (*T* −* T*_0_) × (log2) / (log*N* − log*N*_0_) (insert). The percentage of Ki67-positive cells was determined by flow cytometry after 48 h of seeding (right panel). Growth curve *p* values were determined by a two-way ANOVA with Bonferroni correction and the DT and percentage of Ki67-positive cells using the Student *t* test. *p < 0.05, ***p < 0.001. Error bars denote s.e.m. **c** Cells were stained with Nile red 48 h after vehicle (DMSO, NT), 5-Fu (10 µM), Oxa (10 µM) or FOX (5-Fu + Oxa, 10 µM each) treatments. The number of LDs per cell (lower panel) was obtained by counting red lipid bodies on merged pictures (300 cells per condition) (upper panel) (scale bar = 10 µm). Whiskers denote 1st and 99th percentiles. *P* values were determined by the multiple Student *t* test. ***p < 0.001. **d** Relative *LPCAT2* and *PLIN2* mRNA expression levels at 0, 2, 6, 24 and 48 h after vehicle, 5-Fu, Oxa or FOX treatments. *ACTB* was used as a housekeeping gene to calculate ΔCt. The data are expressed as fold changes calculated with 2^-(ΔCt treatment/ΔCt vehicle)^. The data are the results from three independent experiments. *P* values were determined by two-way ANOVA with Bonferroni correction. *p < 0.05, **p < 0.01, ***p < 0.001. Error bars denote s.e.m. **e**–**g** LD content at 48 h of chemotherapy treatments in (**e**) SW620-Ctl *vs* SW620-lpcat2 cells; (**f**) HT29 cells transiently transfected with sineg vs. silpcat2; (**g**) vehicle (DMSO) vs. TSI-01 (10 µM) co-treatment. Whiskers denote 1st and 99th percentiles. *P* values were determined by the multiple Student *t* test. *p < 0.05, **p < 0.01, ***p < 0.001

To understand the significance of LD accumulation, we first evaluated the potential correlation between LD biogenesis and the capacity of CRC cell lines to proliferate. We ensured that regardless of their proliferation state, low- and high-LD CRC cells kept their differences in LD content. As shown in Fig. 3a, these cells displayed a decrease in the LD proportion between 24 and 72 h that was recovered at cell confluency, and maintained their distinct LD content at each time point (Fig. 3a). We next monitored cell proliferation by Ki67 staining and evaluated doubling times from cell growth curves. Surprisingly, despite a high-LD content, HT29 cells did not proliferate more rapidly than low-LD SW620 cells, as evidenced by doubling times and percentages of Ki67-positive cells (DT = 22.8 ± 1.9 h vs. 44.2 ± 4.9 h, and Ki67^+^ cells ≈ 52% vs. 21% for SW620 and HT29 cells, respectively) (Fig. 3b). The lack of LD impact on cell proliferation was further confirmed by the fact that up- or downregulation of *LPCAT2* or inhibition of LPCAT2 activity did not modify proliferation rates (Supplementary Fig. 4a–c).

Since LDs were shown to accumulate under stress conditions such as nutrient deprivation or hypoxia^17^, we next assessed whether chemotherapy treatment could have any impact on LD production and determined the potential key role of LPCAT2 in these conditions. To that aim, we treated cells with two anticancer drugs commonly used for the treatment of advanced CRC stages, 5-fluorouracil (5-Fu) and oxaliplatin (Oxa) alone or in combination (FOX) for 48 h. LD accumulation was observed in either single or combination drug treatments in both cell lines. In addition, major LD accumulation was observed in high-LD CRC cells and under FOX treatment. It should also be noted that when comparing drugs separately, 5-Fu displayed the highest capacity to trigger LD production (Fig. 3c, lower panel). Interestingly, great heterogeneity within high-LD CRC cells was found upon chemotherapy, some cell populations having more LD than the others (Fig. 3c, upper panel), and also no significant impact on TG levels was found upon drug treatment (Supplementary Fig. 5a). In agreement with enhanced LD production, chemotherapy with 5-FU but not Oxa correlated with increased *LPCAT2* mRNA levels (Fig. 3d, upper panels). In addition, mRNA levels of perilipin 2 (*PLIN2*), a LD coat protein considered as a LD biomarker in metabolically active cells and a key factor for LD biogenesis^20^, was markedly increased in HT29 cells after treatments supporting LD formation (Fig. 3d, lower panels). Upregulation of LPCAT2 expression in low-LD CRC cells (SW620-lpcat2) promoted enhanced LD formation upon 5-Fu and FOX treatments (Fig. 3e; Supplementary Fig. 5b and c) associated with increases in TG cellular content (Supplementary Fig. 5d). Conversely, transient *LPCAT2* silencing (silpcat2) in high-LD CRC cells dramatically decreased chemotherapeutic drug-induced LD accumulation (Fig. 3f; Supplementary Fig. 5e). In addition, selective inhibition of LPCAT2 enzymatic activity with TSI-01 in HT29 cells significantly prevented LD accumulation especially induced by 5-Fu and FOX treatments (Fig. 3g; Supplementary Fig. 5f). To determine whether cell capacity to accumulate LDs was related to chemotherapy concentration, the LD content was assessed in cells treated with increasing FOX concentrations. As shown in Supplementary Fig. 6a and b, FOX concentrations triggering maximum LD accumulation corresponded to maximum FOX-induced cell death in each cell line. Collectively, the results indicate that chemotherapeutic drugs can promote LD production in a LPCAT2-dependent manner.

### LPCAT2-dependent LD biogenesis promotes chemoresistance

**Fig. 4:**
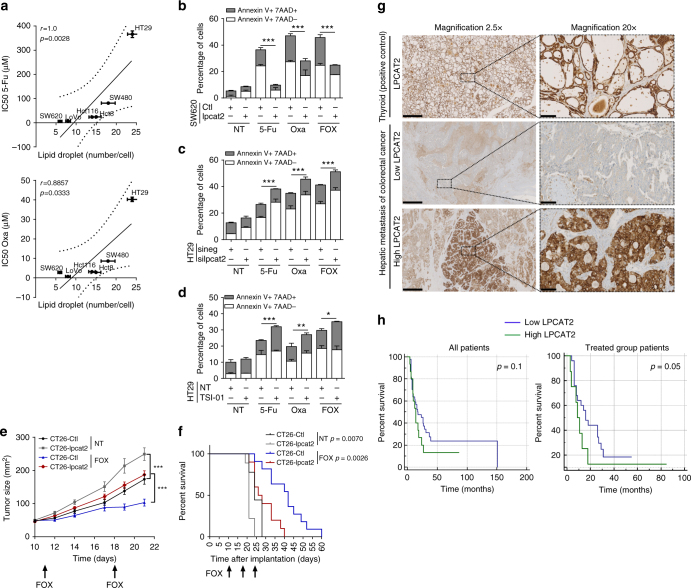
LPCAT2-dependent LD production confers CRC resistance to chemotherapy. **a** Cell viability of CRC cell lines after 48 h of treatment with concentration ranges of 5-Fu and Oxa. IC_50_ for each cell line was compared to the basal LD content and Spearman correlation coefficients were calculated. Error bars denote s.e.m. **b**–**d** Annexin V/7AAD staining after 72 h of treatment with vehicle (NT), 5-Fu, Oxa or FOX in (**b**) SW620-Ctl vs. SW620-lpcat2 cells; (**c**) HT29 cells transiently transfected with sineg vs. silpcat2; (**d**) HT29 cells co-treated or not with TSI-01. The data are mean ± s.e.m. of three independent experiments. *P* values were determined by the multiple Student *t* test. *p < 0.05, **p < 0.01, ***p < 0.001. **e** Eight-week-old female balb/c mice bearing CT26-Ctl or CT26-lpcat2 tumours (*n* = 10 per group) received i.p. FOX injections (5-Fu, 5 mg/kg + Oxa, 6 mg/kg) once a week for 3 weeks. *P* values were determined by a two-way ANOVA with Bonferroni correction. ***p < 0.001. Tumour size in mm^2^ over time is represented as median ± s.em. **f** Kaplan–Meier cumulative survival plots for mice groups described in (**e**), with *p* values assessed by the log-rank test. **g** Histological LPCAT2 staining performed on hepatic metastasis samples from CRC patients (*n* = 79). Representative images of thyroid sections and tumours with low and high-LPCAT2 expression are shown at ×2.5 and ×20 magnification (sections, 1 mm and 100 µm, respectively). **h** Relapse-free survival (RFS) based on LPCAT2 scoring, on all CRC patients (*n* = 79; all patients, left panel) and on those who had received neoadjuvant chemotherapy (*n* = 54; treated group patients, right panel). Kaplan–Meier plots are presented with *p* values assessed by the log-rank test

LD accumulation upon chemotherapy treatments could thus be more likely related to the emergence of CRC cell resistance. Hence, CRC cell line sensitivity toward 5-Fu and Oxa was evaluated and correlated with their basal LD content. A strong positive correlation between 5-Fu and Oxa IC_50_ and LD density was observed, which strengthened the notion that LD accumulation is associated with a poor chemotherapeutic drug response (Fig. 4a; Supplementary Fig. 7a). To further characterise the link between LD overproduction and chemoresistance, we evaluated the impact of LPCAT2 on anticancer drug-induced cell death. Overexpression of LPCAT2 in SW620 cells decreased the percentages of both early (Annexin V+/7AAD−) and late (Annexin V+/7AAD+) apoptotic cells induced either by drugs alone or the FOX combination (Fig. 4b). Conversely, *LPCAT2* silencing or inhibition of LPCAT2 activity sensitised HT29 cells to chemotherapy (Fig. 4c, d). To rule out a direct effect of LPCAT2 on cell death, we evaluated the impact of alternative pathways involved in LD accumulation, such as DGAT2, which supports TG synthesis and LD expansion at ER-LD contact sites^21^. We also checked if supplying cells with oleic acid (OA), a potential fatty acyl donor for acyltransferases, could potentiate the effects of FOX therapy^22^. Neither DGAT2 selective inhibitor (DGAT2i) nor OA alone significantly modified basal HT29 cell LD content; however, they had a non-significant tendency to increase FOX capacity to promote droplet accumulation (Supplementary Fig. 7b). Surprisingly, DGAT2i was found to increase HT29 cell FOX resistance while OA moderately decreased it (Supplementary Fig. 7c). We also transiently downregulated *PLIN2* in HT29 cells (Supplementary Fig. 8a) and found that it sharply blocked basal and chemotherapy-induced LD production (Supplementary Fig. 8b) and restored sensitivity to 5-Fu and Oxa (Supplementary Fig. 8c). In light of these results and the study of Qiu et al. reporting that, in clear-cell renal cell carcinoma, HIF2α supported PLIN2 expression and LD production associated with ER homeostasis during cellular stress^5^, we next explored the likely contribution of HIF2α in our models. Analysis of the HIF2α gene (*EPAS1*) and the protein expression profile in CRC cell lines showed that HT29 cells, despite a high-LD content, do not express high HIF2α protein levels, which could be related to the high degree of post-translational modifications of this transcription factor^23^ (Supplementary Fig. 9a and b). Hence, the data tends to suggest that HIF2α might not be involved in LD accumulation in basal conditions, which was further confirmed in downregulation experiments. *EPAS1* downregulation slightly increased *PLIN2* mRNA levels and protein expression, but did not impact LPCAT2 expression (Supplementary Fig. 9c and d), LD content (Supplementary Fig. 9e) or cell sensitivity to FOX therapy (Supplementary Fig. 9f). Combining results thus confirmed that LD production supported by LPCAT2 is involved in chemoresistance.

To evaluate the impact of LPCAT2-mediated LD production on tumour cell growth in vivo, we generated a mouse CRC cell line (CT26) overexpressing or not LPCAT2 (Supplementary Fig. 10a). We controlled that constitutive expression of LPCAT2 in these cells resulted in enhanced LD production and did not alter cell growth in vitro as seen with the SW620-lpcat2 model (Supplementary Fig. 10b and c). We further extended in vitro observations in a syngeneic model of CT26-lpcat2 or CT26-Ctl tumours subcutaneously injected into balb/c mice that we challenged with three intraperitoneal injections of FOX or vehicle. In contrast to in vitro experiments, significant enhanced tumour progression was seen in CT26-lpcat2 tumour-bearing mice. CT26-lpcat2 tumour grafts responded partially to FOX therapy compared to control grafts (Fig. 4e) and were more aggressive, as evidenced by reduced survival in these mice (Fig. 4f). The role of LPCAT2 was further confirmed by shRNA-mediated stable knockdown of *Lpcat2* in vivo. CT26 cells, stably transduced by control shRNA (shneg) or four different shRNA targeting the *Lpcat2* gene (sh*Lpcat2* #1, #2, #3 and #4), were generated (Supplementary Fig. 10d and e) and two of them injected subcutaneously in balb/c mice. *Lpcat2* knockdown (sh*Lpcat2* #2 and #4) resulted in a slight reduction in tumour progression under FOX therapy as compared to shneg tumour-bearing mice (Supplementary Fig. 10f), but was associated with significantly increased mouse survival (Supplementary Fig. 10g).

To assess the potential clinical relevance of these data, we performed immunohistochemical (IHC) staining of LPCAT2 on liver metastasis samples from 79 CRC patients (Supplementary Table 2). LPCAT2 IHC staining was heterogeneous but discriminated between low- and high-LPCAT2 samples (Fig. 4g). On the basis of LPCAT2 IHC scoring, we next evaluated relapse-free survival (RFS) in all patients or in CRC patients who received neoadjuvant therapy. The RFS rate did not seem to be impacted by LPCAT2 overexpression in all patients (Fig. 4h, left panel) but was reduced in metastatic patients who received neoadjuvant therapy (Fig. 4h, right panel). As a whole, for the first time in preclinical studies these data show a correlation between LPCAT2 expression, LD production and resistance to chemotherapy.

### LPCAT2-induced LD accumulation supports ER homeostasis

**Fig. 5:**
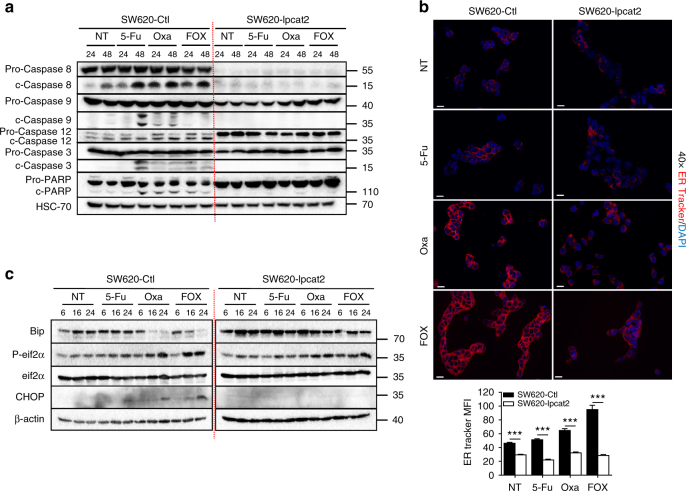
LPCAT2-induced LD accumulation blunts chemotherapy-induced ER stress. **a** Impact of LPCAT2 on chemotherapy-induced cell death pathways in SW620-lpcat2 vs. SW620-Ctl cells after 24 and 48 h of treatments. A representative blot of pro- and cleaved forms of caspases 8, 9, 12, 3 and PARP is shown from three independent experiments. HSC-70 was used as loading control. **b** ER tracker staining was performed 24 h after chemotherapy treatments. Representative images of three independent experiments are shown (×40 magnification, scale bar = 20 µm) (upper panel). ER tracker median of fluorescence intensity (MFI) was calculated in three independent experiments (300 cells per condition) (lower panel). *P* values were determined by the multiple Student *t* test. ***p < 0.001. Error bars denote s.e.m. **c** Western-blot analysis of ER stress markers Bip, CHOP, P-eif2α and total eif2α in SW620-lpcat2 and SW620-Ctl cells after 6, 16 and 24 h of chemotherapy treatments. β-actin was used as loading control

We next explored the impact of LPCAT2 overexpression on key markers of cell death pathways induced by 5-Fu, Oxa or FOX treatments. As shown in Fig. 5a, constitutive expression of LPCAT2 in SW620 cells curtailed chemotherapy-induced activation of all caspases involved in extrinsic (caspase 8), intrinsic (caspase 9) and ER stress (caspase-12) pathways and of enzymes corresponding to the end of the caspase cascade, i.e., caspase 3 and poly(ADP-ribose) polymerase (PARP). Since caspase-12 cleavage/activation was blunted in SW620-lpcat2 cells, we hypothesised that LPCAT2 overexpression could hence impair chemotherapy-induced ER stress. A common feature of cells undergoing ER stress is enlargement of the ER lumen, which can be monitored by increased ER-tracker probe uptake and enhanced MFI^24^. Overexpression of LPCAT2 was associated with a reduction in ER-tracker MFI upon chemotherapy treatments, suggesting an alteration in ER stress pathway induction (Fig. 5b). This was further reinforced by immunostaining cells with the ER stress marker protein disulfide isomerase (PDI). Hence, increased expression of PDI was only found in cells with low LPCAT2 expression, strengthening the notion that overexpression of LPCAT2 reduces chemotherapy’s capacity to trigger ER stress (Supplementary Fig. 11). Moreover, analysis of ER stress marker expression showed that Oxa and FOX treatments activated eif2α and CHOP in SW620-Ctl, whereas decreased phosphorylation of eif2α and loss of CHOP induction was found in LPCAT2-overexpressing cells (Fig. 5c). As well, the combined decrease in the Bip protein level and CHOP accumulation in SW620-Ctl cells indicated commitment cell death, and conversely the maintenance of Bip expression and loss of CHOP expression argued in favour of cell death escape in SW620-lpcat2 cells (Fig. 5c).

**Fig. 6:**
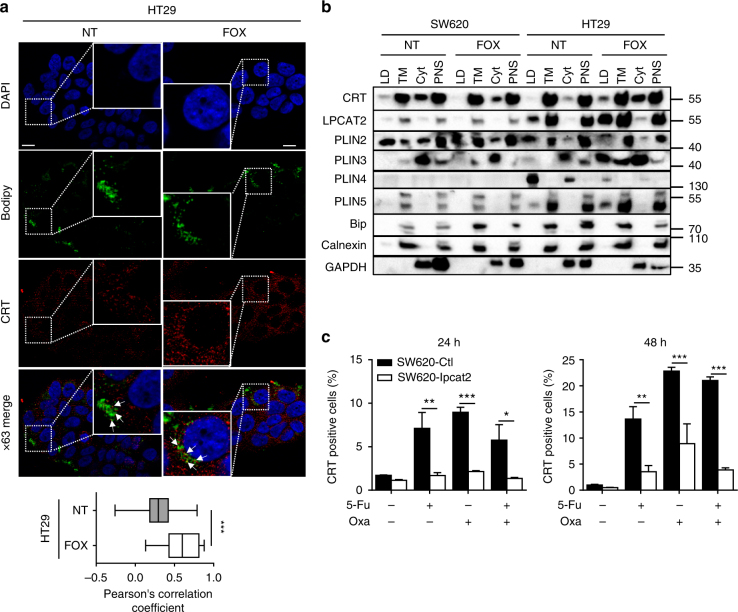
LPCAT2-induced LD accumulation blunts ecto-CRT exposure. **a** HT29 cells were treated for 48 h with or without FOX and then stained with Bodipy 493/503 (green) and CRT antibody (FMC75) conjugated with goat anti-mouse Alexa568 antibody (red) (upper panel). The Pearson correlation coefficient was calculated between CRT and Bodipy fluorescences (lower panel). **b** LDs were isolated from confluent cells treated or not with FOX for 48 h. A representative blot of compartment markers of LDs, total membrane (TM), cytosol (cyt) and post-nuclear supernatant (PNS) fractions is shown from three independent experiments. **c** Cell-surface CRT immunostaining after 24 and 48 h of chemotherapy treatments. Data represent CRT-positive cells in the DAPI-negative fraction. Data are mean ± s.e.m. of three independent experiments. *P* values were determined by the multiple Student *t* test. *p < 0.05, **p < 0.01, ***p < 0.001. Error bars denote s.e.m

CRT, a soluble ER Ca^2+^-binding protein and ER chaperone, can be translocated from ER to plasma membrane of apoptotic dying cells generally after ER-stress-induced phosphorylation of eif2α^25^. We therefore hypothesised that the weak capacity of chemotherapy to induce ER stress in high-LD cells might be associated with an impairment of CRT membrane exposure (ecto-CRT), probably because of CRT sequestration by LDs. Combined CRT and LD staining in HT29 cells showed that CRT was localised in LDs either with or without chemotherapy (Fig. 6a). To confirm this hypothesis, we isolated LD from SW620 and HT29 cells treated or not treated with FOX and assessed CRT distribution across fractions. LD fraction purity is generally controlled by the expression levels of perilipin family members (PLIN2 notably); however, perilipins have been reported to be differentially expressed depending on cell types and to have variable subcellular locations depending on the cell’s metabolic status^26^. We first explored the expression profile of perilipins in CRC cell lines and showed that PLIN2 was the major perilipin present in SW620 cells, whereas HT29 cells expressed more PLIN3, 4 and 5; however, no PLIN1 protein expression was detected in all cell lines (Supplementary Fig. 12a and b). Hence, PLIN2, 3, 4 and 5 were used as LD markers in fractionation experiments, and fraction purity was further confirmed by TG quantification (Supplementary Fig. 13). As shown in Fig. 6b, an enrichment of CRT in the LD fraction was only seen in FOX-treated HT29 cells. Remarkably, LPCAT2 overexpression in SW620 cells triggered a strong decrease in ecto-CRT (Fig. 6c) and inhibition of LPCAT2 activity in HT29 cells weakly but significantly potentiated FOX-induced plasma membrane CRT expression (Supplementary Fig. 14a).

### Inhibition of LD biogenesis reverses the resistance phenotype

**Fig. 7:**
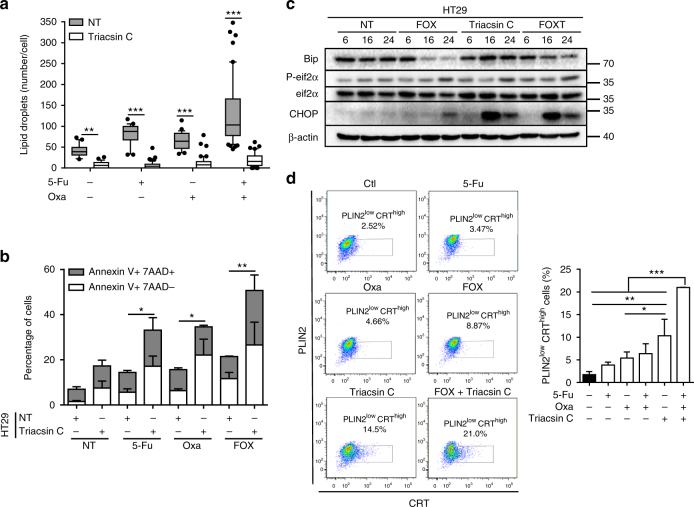
Inhibition of LD production restores chemotherapy-induced ER stress. **a** Nile red LD staining in HT29 cells treated with chemotherapy in the absence (NT) or presence of triacsin C (10 µM). Mean number of LDs /cell with whiskers denoting 1st and 99th percentiles. *P* values were determined by the multiple Student *t* test. *p < 0.05, **p < 0.01, ***p < 0.001. **b** Impact of pharmacological inhibition of LD production on HT29 cell viability after 72 h of treatments. The data are mean ± s.e.m. of three independent experiments. *P* values were determined by the multiple Student *t* test. *p < 0.05, **p < 0.01. **c** Representative blot of ER stress marker expression from HT29 cells treated or not with FOX, in the absence (FOX) or presence of triacsin C (FOXT) from three independent experiments. β-actin was used as the loading control. **d** Left panel, representative pseudocolor flow cytometry plots with gating strategy. Gating was performed on DAPI-negative cells with low expression of the LD marker PLIN2 (PLIN2^low^) and with high CRT plasma membrane expression (CRT^high^). Right panel, mean percentages ± s.e.m. of PLIN2^low^ and CRT^high^ cells corresponding to three independent experiments. *P* values were determined by two-way ANOVA with Bonferroni correction. *p < 0.05, **p < 0.01, ***p < 0.001

**Fig. 8:**
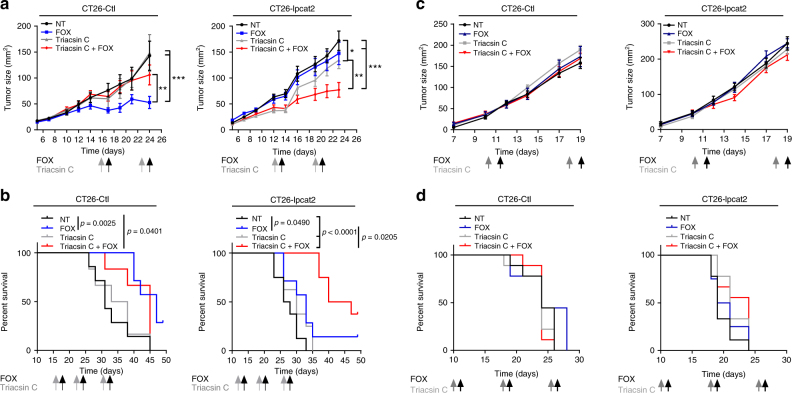
Inhibition of LD production promotes tumour regression. **a** Twelve-week-old female balb/c mice (*n* = 32) bearing CT26-Ctl or CT26-lpcat2 tumours were subdivided into four groups (*n* = 8 per group) and intraperitoneally injected with triacsin C (2 mg/kg) once a week for 3 weeks followed the next day by FOX, or with FOX and triacsin C alone or with vehicles alone (NT groups). Tumour size in mm^2^ over time is represented as median ± s.e.m. *P* values were determined by two-way ANOVA with Bonferroni correction. *p < 0.05, **p < 0.01, ***p < 0.001. **b** Kaplan–Meier plots with time of death as endpoint for mice described in **a** are presented with *p* values assessed by a log-rank test. **c** Twelve-week-old female NMRI-nude mice (*n* = 32) bearing CT26-Ctl (left panels) or CT26-lpcat2 (right panels) tumours were subdivided into four groups (*n* = 8 per group) and treated as described in **a**. **d** Kaplan–Meier plots with time of death as endpoint for mice described in **c** are presented with *p* values assessed by the log-rank test

Since LD expansion occurs when excess lipids are esterified into TGs and requires additional phospholipid production to coat the enlarging surface^9^, we used triacsin C, a long-chain fatty acyl CoA synthetase inhibitor^27^, to counteract neutral lipid and phospholipid synthesis and subsequently LD biogenesis. As expected, triacsin C remarkably curtailed the accumulation of LD induced by anticancer drugs (Fig. 7a and Supplementary Fig. 14b). This collapse in LD production allowed the reversion of high-LD HT29 cell resistance to chemotherapy (Fig. 7b), which was related to ER-stress pathway activation (Fig. 7c) and to substantial increases in the proportion of cells with low-LD content (PLIN2^low^) and high CRT membrane exposure (CRT^high^) (Fig. 7d). Additionally, triacsin C also remarkably increased 5-Fu’s capacity to promote CRT translocation (Supplementary Fig. 14c). The data, thus, suggest that expression of ecto-CRT was dependent on chemotherapy’s capacity to induce LD accumulation. To confirm that, we tested the capacity of the well-known ecto-CRT inducer mitoxantrone^28^ to accumulate LD. We found that at the same cytotoxic concentration as 5-Fu or Oxa (Supplementary Fig. 14d), mitoxantrone did not significantly impact LD accumulation in SW620 cells, which correlated with the induction of a large proportion of cells harbouring ecto-CRT. Besides, in HT29 cells, mitoxantrone-induced LD accumulation resulted in less CRT membrane exposure (Supplementary Fig. 14e and f). Reversion of the chemoresistance phenotype through the modulation of LD biogenesis was further reinforced in vivo in mice. CT26-Ctl and CT26-lpcat2 tumour-bearing balb/c mice were injected intraperitoneally with triacsin C the day before FOX injection. We observed that triacsin C drastically enhanced the antitumoural effects of FOX on CT26-lpcat2 tumour-bearing mice and improved their survival (Fig. 8a and b, right panels), although it did not impact FOX potential in CT26-Ctl tumour-bearing mice groups (Fig. 8a, b, left panels). We also found that the antitumoural effect of FOX was completely lost in immunodeficient mice bearing CT26-Ctl tumours (Fig. 8c, d, left panels) and that triacsin C’s capacity to potentiate chemotherapy was abrogated in immunodeficient mice bearing CT26-lpcat2 tumours (Fig. 8c, d, right panels), suggesting that the immune system plays a crucial role.

Collectively, the data underline the intimate relationship existing between LD biogenesis, modulation of ER stress-mediated CRT exposure, immune surveillance and tumour progression despite a chemotherapy regimen.

### LD-induced CRT sequestration curtails tumour immunogenicity

**Fig. 9:**
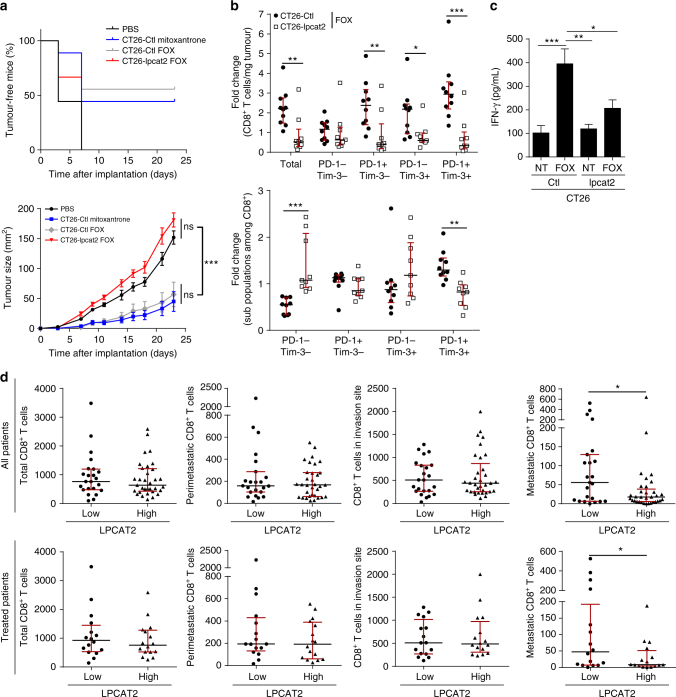
LPCAT2-induced LD accumulation prevents ICD. **a** Vaccination experiments: upper panel, percentage over time of tumour-free mice for each group. Lower panel, growth curves over time of setting tumours (*n* = 9 of 8-week-old female balb/c mice per group). Values are mean ± s.e.m. *P* values were determined by two-way ANOVA with Bonferroni correction. *p < 0.05, **p < 0.01, ***p < 0.001. **b** CT26-lpcat2 (*n* = 20) and CT26-Ctl (*n* = 20) tumour-bearing 8-week-old female balb/c mice were subdivided into two groups (*n* = 10 per group) and intraperitoneally injected with vehicle or FOX. Tumour grafts were immunostained for CD3^+^CD8^+^ T-cell-subtype identification and PD-1 and Tim-3 subpopulation phenotyping. Fold change of the number of CD8^+^ T cells and percentage of sub-populations among CD8^+^ cells were calculated for each condition according to their respective untreated group. *P* values were determined by the multiple Student *t* test. *p < 0.05, **p < 0.01, ***p < 0.001. The data represent medians with interquartile ranges. **c** Supernatants of dissociated tumours described in **b**) were assayed by ELISA for IFN-γ secretion. *P* values were determined by two-way ANOVA with Bonferroni correction. *p < 0.05, **p < 0.01, ***p < 0.001. Error bars denote s.e.m. **d** Histological LPCAT2 and CD8 immunostainings performed on hepatic metastasis samples from 56 CRC patients. Absolute quantification of CD8^+^ T cells in the metastatic, peri-metastatic, invasion and total site was performed and compared between groups with low and high-LPCAT2 expression/scoring. Comparisons were made for all CRC patients (*n* = 56) and for patients who received neoadjuvant chemotherapy (*n* = 32). *P* values were determined by Mann–Whitney *U* test. *p < 0.05

Cell-surface exposure of CRT on dying tumour cells was reported to support the early events of ICD, a mechanism that can be triggered by chemotherapeutic drugs, although not all, which contributes to efficient tumour recognition and elimination by the immune system^28,29^. Given that CRT exposure was hampered by LPCAT2-mediated LD accumulation, we hypothesised that immunogenicity of high-LD dying CRC cells could be impaired and contribute to chemoresistance. Hence, the capacity of CRC cells overexpressing LPCAT2 to induce ICD in vivo was assessed in vaccination experiments according to the guidelines of Kepp et al^30^. Mice were subcutaneously vaccinated with PBS (negative control), CT26-Ctl cells treated with mitoxantrone (an ICD inducer used as a positive control), FOX-treated CT26-Ctl or FOX-treated CT26-lpcat2 cells and challenged 1 week after the first injections with CT26-Ctl cells whose growth had been monitored. About 50% of the mice were vaccinated by injection of mitoxantrone or FOX-treated CT26-Ctl cells, while no mice were vaccinated with CT26-lpcat2 cells treated with FOX (Fig. 9a). In the setting of growing FOX-treated tumours, we further evaluated changes in the composition of tumour immune infiltrate. We notably focused on CD3^+^CD8^+^ T-cell infiltrate, which was shown to predict positive therapeutic response after chemotherapy^31,32^. As expected, CT26-Ctl tumour-bearing mice treated with FOX presented more CD8^+^ tumour infiltration 1 week after FOX injection compared to untreated mice, but FOX treatment failed to increase CD8^+^ T-cell infiltration in CT26-lpcat2 tumour grafts (Fig. 9b, upper panel). These results prompted us to assess the activation/exhaustion status of infiltrated CD8^+^ T cells. We analysed the frequency of CD8^+^ T cells expressing both programmed cell death-1 (PD-1) and T-cell immunoglobulin and mucin-domain containing-3 (Tim-3), two markers of T-cell activation/exhaustion^33^ (Supplementary Fig. 15d). The FOX regimen induced TIM3^+^ PD1^+^ subpopulation accumulation in control grafts as compared to LPCAT2-overexpressing grafts, thus suggesting that chemotherapy only activates a CD8^+^ immune response in control tumours (Fig. 9b, lower panel). This was reinforced by the fact that FOX could only stimulate IFN-γ production in CT26-Ctl grafts (Fig. 9c). To consolidate the role of LPCAT2 overexpression/LD production on CD8^+^ T-cell exhaustion, LPCAT2 and CD8 IHC staining was performed on liver metastasis samples from CRC patients. IHC analysis showed that LPCAT2 overexpression was associated with weak CD8^+^ T-cell infiltration only at the metastatic site (Fig. 9d).

**Fig. 10:**
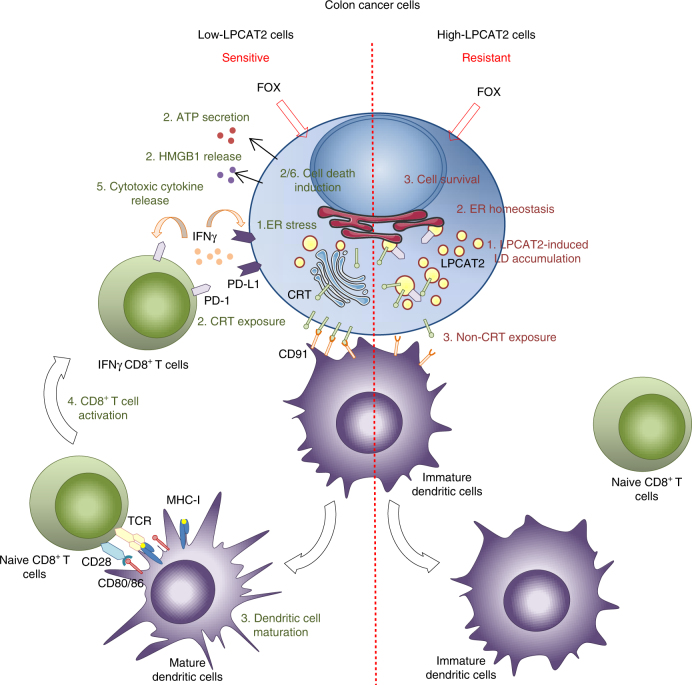
Suggested model for chemotherapy-resistant CRC phenotype mediated by LPCAT2 overexpression. Contrary to sensitive cells, FOX treatment in high-LPCAT2 cells promotes LD accumulation leading to 1) ER homeostasis, 2) CRT sequestration into LD. The resulting failure in DC maturation leads to limited recruitment/activation of naïve CD8^+^ T cells via co-stimulatory factors CD80/86/MHC-I and CD28/TCR. Impaired ICD associated with reduced recruitment of IFNγ-secreting CD8^+^ T cells to tumour site could consequently avoid PD-L1 and PD-1 exposure on tumour and CD8^+^ T cells, respectively, and thus may lead to immunotherapy failure in addition to FOX resistance. HMGB1 High-mobility group box, IFN-γ interferon gamma, PD-1 programmed cell death-1, PD-L1 programmed cell death ligand-1, TCR T-cell receptor, MHC-I major histocompatibility complex-I

Collectively, these data highlight a yet undescribed direct role of LD accumulation in CRC cell chemoresistance and suggest a new model in which the LPCAT2 expression profile could discriminate between responsive and resistant tumour cells. In this model, the high-LPCAT2 cancer cell phenotype under anticancer agent treatments such as the FOX regimen, produces more LDs to maintain ER homeostasis, sequester CRT and impair ICD (Fig. 10).

## Discussion

Despite an increasing number of reports highlighting the role of LD accumulation in tumour progression and cancer aggressiveness^5,7,16^, few studies have investigated the role of these intracellular lipid bodies in tumour resistance to chemotherapy, which is a major obstacle for treatment success in cancer patients. Indeed, LD accumulation was found to accumulate in some drug-resistant cell lines^34–37^. However, no direct links or full comprehensive mechanisms have been provided so far. It therefore seemed pertinent to better understand the significance of LD accumulation in the context of chemotherapeutic treatments in colorectal cancer (CRC). Herein, for the first time through integrated approaches, we provide a mechanism linking LD accumulation and resistance to conventional CRC chemotherapies such as FOX, involving the acyltransferase LPCAT2.

In agreement with altered phosphatidylcholine (PC) metabolism in many types of cancer including CRC, overexpression of key enzymes of PC metabolism/remodelling, particularly CKα and LPCAT1, were found to correlate with cancer progression. LPCAT2 was recently associated with prostate cancer aggressiveness^38–42^. Interestingly, these enzymes participate, at different levels, in the regulation of LD formation, notably the Lands cycle acyltransferases LPCAT1 and LPCAT2, which play a key role in LD membrane synthesis and expansion through PC biosynthesis^8,10,19,43^. We showed that only LPCAT2 was correlated with basal LD content in CRC cell lines and supported LD formation, as evidenced by manipulation of LPCAT2 expression levels and enzymatic activity in high- and low-LD CRC cells. Our data from co-localisation/LD isolation experiments also confirmed the location of LPCAT2 in the LD monolayer in LD-rich cells^11^. The apparent LPCAT2 self-sufficiency in basal LD production could be explained by the fact that the Lands cycle can be fuelled with acyl-CoA and lysophosphatidylcholine (LPC) by the activities of ACSL3 and PLA2 enzymes, respectively^44^, and that LD-localised PC production over LD is crucial to prevent LD coalescence^10^. In addition, LD expansion supported by TG production could be sustained by phospholipid synthesis to avoid an imbalanced volume-to-surface ratio of droplets. In our models, DGAT2, an end enzyme of the glycerol 3-phosphate pathway contributing to TG synthesis, did not seem involved in basal LD production, while LPCAT2-overexpressing cells accumulated TG during chemotherapy. These data suggest a possible alternative route supporting TG and LPC production, potentially through PC and diacylglycerol (DG) transacylations, as already evidenced in plants (through the phospholipid:diacylglycerol acyltransferase (PDAT) enzyme) and yeast (through the lrop 1 enzyme)^44^, or may reflect modifications in the lipid neutral core composition such as a TG to the cholesterol-ester switch or modifications in TG fatty-acyl composition, since moderate potentiation of FOX-induced cell death was observed when supplying cells with oleic acid. Future studies that characterise LD content under chemotherapy may help clarify these disparities^45^. Likewise, LPCAT2 and PLIN2 also supported LD formation upon FOX and mainly 5-Fu treatments, both in low-(SW620) and high-LD HT29 cells, although not to the same extent. These results indicate that each CRC cell line had different capacity in producing LD and in accumulating LD. It was also quite surprising not to have found a correlation between LPCAT2-dependent LD production and CRC cell growth since it was shown that LD production supports cell proliferation^12,16,35^. Nevertheless, we observed that neither cell line had the same proliferative capacity and that LD content tended to decrease during the cell’s proliferative phase, suggesting a possible use of LD for cell division. In addition, lipid accumulation has been recognised as a preventive mechanism of lipid storage in case of nutrient stress so as to supply cells with sufficient energy for their survival through lipid β-oxidation^5,46,47^. A possible explanation for these observations, not addressed in this study, is that LD lipolysis may be more prominent in highly proliferating cells or that cells may utilise other lipid/nutrient sources to sustain proliferation, while LDs are allocated to other functions in high-LD cells. Relocation of PLIN2, PLIN5 and PLIN3 to the LD fraction in high-LD cells during FOX treatment, could argue in favour of LD protection against lipophagy and lipolysis, by the inhibition of lipase activities such as ATGL^48^.

As evidenced by others^18,35,47^, we found great heterogeneity in LD density within the same cell line, in particular in HT29 cells, both at resting and under chemotherapy treatments, which underlines the possible role of LD accumulation in fuelling the surrounding cells with lipids and in limiting genotoxic stress. Indeed, it was suggested that high-LD cells within a same population may contribute to the sequestration of damaging molecules such as reactive oxygen species (ROS) or lipid peroxides, and may thus protect whole cell populations from cytotoxic stress^5,15,46,49^.

According to the central role of caspase-12 in cell death-mediated activation of the ER stress pathway through the unfolded-protein response (UPR)^50^ and to the recently described role of PLIN2-induced LD accumulation in maintaining ER homeostasis^5^, we found that the chemotherapy-induced ER stress pathway was profoundly impaired in high-LD HT29 cells. Maintenance of ER homeostasis in high-LD cells could be explained by a higher capacity of these cells to handle ROS/lipotoxicity injuries or to limit excessive ER Ca^2+^ release and subsequent ER stress-mediated activation of caspase-12 and initiation of cell death^51,52^. Hence, disruption of LD homeostasis may also contribute to both cell phenotypes, which would deserve further investigation. Nevertheless, chemotherapy-induced LPCAT2/LD accumulation appeared critical for CRC cells’ response to treatment. Indeed, increases in LD density triggered by anticancer drugs were associated in vitro with cell survival and inhibition of caspase activation and, in vivo, by enhanced tumour progression. Consequently, LD directly supports resistance and LPCAT2 overexpression emphasises LD production in these phenotypes.

Maintenance of ER integrity and impairment of chemotherapy-induced cell death both contribute to limiting danger-associated molecular pattern (DAMP) exposure. We thus showed in cells with high-LD content and high-LPCAT2 expression, that plasma membrane CRT exposure, which is an early event of ICD, was impaired due to 1) the incapacity of cells to induce a sustained UPR, especially through the phosphorylation of eif2α^25,53^; 2) the translocation of CRT from ER to LD instead of Golgi to membrane translocation^54^, which confirmed the work of Turro et al. demonstrating the presence of CRT in the LD fraction by mass spectrometry^55^. Panaretakis and colleagues have shown that caspase 8 is essential for CRT exposure, which supports the fact that LPCAT2 overexpressing cells with a partial loss of caspase 8 proform expose three to four times less CRT to plasma membrane^54^.

Impairment of CRT membrane exposure is associated with the incapacity of dendritic cells (DCs) to recognise the “eat me” signal of dying tumour cells that are required for phagocytosis and antigen uptake, leading to DC maturation and cross-priming of CD8^+^ T cells. This activation transduces signals for infiltrated-CD8^+^ T lymphocytes to migrate to the tumour site and produce cytotoxic cytokines such as IFN-γ^28,29,56^. We show herein for the first time that overexpression of LPCAT2 turns CRC cells into non-immunogenic ones after FOX treatment, which was supported by a decrease in activated IFN-γ producing-CD8^+^ T-cell infiltration in vivo in mice. Moreover, the fact that tumour growth was potentiated by LPCAT2 overexpression in basal conditions strengthened the role for immune tumour microenvironment modulation in tumour expansion. This is further reinforced by previous studies showing that COX-2 is located in LDs and contributes to PGE_2_ production in CRC cells^16^, which could provide an inflammatory environment leading to immunosuppressive mechanisms^57^. More importantly, discrimination between high- and low-LPCAT2 patients confirmed a differential response to treatment and impact on the survival rate, potentially through the modulation of CD8^+^ T-cell infiltration. Altogether, these data provide the first evidence that LPCAT2 IHC scoring or analysis of tissue LD content by Raman spectroscopy, for instance, could be used as potential prognostic factors for early stages of CRC, as well as potential predictive factors of the patient’s response to conventional neoadjuvant therapies or to the more recently described immunotherapies in advanced stages^58,59^. Moreover, further studies in larger patient cohorts would be required to fully assess the prognostic and predictive value of LPCAT2/LD considering tumour genetics.

## Methods

### Cell culture and viability assays

Human colorectal cancer cell lines SW620, LoVo, Hct116, Hct8, SW480, HT29 and murine colorectal cancer CT26 were obtained from the American Type Culture Collection. Cells were maintained in a 5% CO_2_ humidified atmosphere at 37 °C and cultured in DMEM or RPMI-1640 supplemented with 10% FBS (Dutscher). Cells were routinely tested for mycoplasma contamination using the Mycoalert Mycoplasma Detection Kit (Lonza).

Treatments were carried out for 6, 16, 24, 48 or 72 h with 10 µM of 5-fluorouracil (Sigma Aldrich), oxaliplatin (Accord Healthcare Limited), triacsin C (Cayman Chemical) LPCAT2 inhibitor: TSI-01 (Cayman Chemical), DGAT2 inhibitor: PF-06424439 (Sigma Aldrich), or oleic acid (Sigma Aldrich). Cell viability was determined using the annexin V-FITC and 7-aminoactinomycin D (7AAD) staining from BD Biosciences according to the manufacturer’s instructions. The inhibitory concentrations 50% (IC_50_) were assessed by crystal violet staining after 48 h of treatment. IC_50_ values were calculated by a four-parameter non-linear regression with SigmaPlot version 6 software (Systat software, Inc.).

### Animal studies

Female balb/c or NMRI-Nude mice about 6 to 12 weeks old were purchased from Janvier Labs. All mice were housed and maintained in a designated pathogen-free area accredited by the Federation of Laboratory Animal Science Associations (FELASA) in accordance with the University of Burgundy Animal Experimental Ethics Committee guidelines.

To induce tumour formation, 4×10^5^ CT26-Ctl or CT26-lpcat2 or CT26 transduced with scrambled shRNA or sh*Lpcat2*#2 or #4 in RPMI were injected subcutaneously into the left flank of 8- or 12-week-old female balb/c or NMRI-Nude mice. Tumour size was monitored three times a week by caliper measurements of the widest diameter and the narrowest diameter. Time of death was considered when mice reached experimental endpoints related to graft size, animal weight and general behaviour. For all animal studies, eight to ten mice per group were used. After tumours reached 50 mm^2^, animals were randomised according to tumour size before any treatment to ensure group homogeneity. All animals were included in the analyses. After randomisation, depending on the groups, mice received an intra-peritoneal injection of glucose 2.5% or FOX (50 mg/kg of 5-fluorouracil and 6 mg/kg of oxaliplatin, Accord Healthcare Limited) once a week for 3 weeks. For combination experiments, 2 mg/kg of triacsin C (Cayman Chemical; resuspended in DMSO: PBS 1 × 1:2) or corresponding vehicle was injected the day before FOX injection.

### Vaccination experiments

CT26-Ctl or CT26-lpcat2 cells were treated for 6 h with a combination of 5-fluorouracil and oxaliplatin (20 µM each). Positive control consisted in CT26-Ctl cells treated with 1 µM of mitoxantrone for 6 h. Sixteen hours after treatments, PBS (negative control) or cells (5×10^5^ cells) were injected subcutaneously into the right flank of 8-week-old female balb/c mice. 6 days later, untreated CT26-Ctl cells (5×10^5^ cells) were implanted subcutaneously in the left flank of mice. Tumour setting and tumour growth were monitored three times a week.

### Patient samples

Hepatic metastasis samples from colorectal cancer patients were obtained from the Georges François Leclerc Cancer Centre (Dijon, France). All patients gave informed consent approved by the local Ethics Committee of the Georges François Leclerc Centre. Review of the pathology reports confirmed the diagnosis. All patients comprised 79 paraffin formalin-fixed paraffin-embedded slides. The treated group comprised patients who received neoadjuvant chemotherapy. All slides were used for relapse-free survival analysis. Fifty-six slides were used for immunohistochemistry CD8/LPCAT2 comparison. The clinical and pathological characteristics of patients are presented in Supplementary Table 2.

### Plasmids, retrovirus, lentivirus production and viral transduction

Retroviral vectors pCMV6 expressing human *LPCAT2* (h-*LPCAT2*; NM_017839) and murine *Lpcat2*(m-*Lpcat2*; NM_173014) were obtained from Origen. Retroviruses were produced by transfecting Phoenix-AMPHO cells (ATCC; CRL-3213) with the indicated expression plasmids using the JetPEI reagent according to the manufacturer’s instructions (Polyplus). Viruses were collected 48 and 72 h after transfection. SW620 cells for human LPCAT2 expression or CT26 cells for murine LPCAT2 overexpression were then incubated with virus-containing medium and 8 µg/mL polybrene (Merck Millipore) for 4 h. After 48 h of recovery in fresh medium, transduced cells were selected with Neomycin (Sigma Aldrich).

Four unique 29mer shRNA constructs in lentiviral GFP vector were obtained from Origen with the following sequences: sh*Lpcat2*#1: 5′-TTCGTCCAGCAGACTACGATCAGTGCCTC-3′; sh*Lpcat2*#2: 5′-ACCTGGCAAGGCTATACATTCCTCCAGCT-3′; sh*Lpcat2*#3: 5′-CTGTCAACTCTTCACGAAGGTGGAGATTG-3′; sh*Lpcat2*#4: 5′-GCTTCTCTTGGAGTGCCTGACCTTAATGT-3′. Lentiviruses were produced by transfecting 293T cells with pGFP-C-shlenti *Lpcat2* or with a scrambled negative control containing a non-effective shRNA cassette associated with a pCMV-VSV-G expression vector and a psPAX2 vector, using the jetPEI reagent according to the manufacturer’s instructions (Polyplus). Viruses were harvested 48 and 72 h after transfection. CT26 cells were then incubated with virus-containing medium and 8 µg/mL polybrene (Merck Millipore) for 4 h. After 48 h of recovery in fresh medium, transduced cells were selected with puromycin (Sigma Aldrich).

### Transient transfection

For small-interfering RNA (siRNA)-mediated knockdown of the *LPCAT2*, *PLIN2* and *EPAS1* (gene encoding HIF2α protein), the cells were reverse-transfected with 10 nM of either the targeting siRNA Silencer® Select or negative control siRNA (Ambion) using Lipofectamine RNAiMax as the transfection reagent (Invitrogen) for 24 h. Fresh or chemotherapy-containing media were then added to cells for 48 or 72 h before subsequent analysis. The sequence of siRNA targeting human *LPCAT2*, *PLIN2* and *EPAS1* are as follows: *LPCAT2* sense 5′-CAACAUACCUAGACCUCCAtt-3′; antisense 5′-UGGAGGUCUAGGUAUGUUGta-3′; *PLIN2* sense 5′-GGGUUAAAGAAGCUAAGCAtt-3′; antisense 5′-UGCUUAGCUUCUUUAACCCtg-3′; *EPAS1* sense 5′-CACCUACUGUGAUGACAGAtt -3′; antisense 5′-UCUGUCAUCACAGUAGGUGaa -3′.

### RNA extraction and quantitative PCR analysis

Total cellular RNA was extracted with TRIzol^®^ RNA Isolation Reagent (Ambion). RNA (300 ng) was reverse-transcribed into cDNA using M-MLV reverse transcriptase, random primers and RNAseOUT inhibitor (Invitrogen). cDNA was quantified by real-time PCR with the Power SYBR Green PCR Master mix (Applied Biosystems; Warrington, UK) on a 7500 Fast Real-Time PCR detection system (Applied Biosystems). Relative mRNA levels were determined by the ΔΔCt method and normalised to the expression levels of human or mouse *Actb*. The primer sequences used are listed in Supplementary Table 3.

### Quantitative analysis of PC and LPC species by HPLC-MS/MS

Total lipids were extracted from cells according to the Folch method^60^. The 19:0 lysophosphatidylcholine (LPC, 10 ng) and 21:0/21:0 phosphatidylcholine (PC, 1 µg) (Avanti Polar Lipids) were used as internal standards. The organic phase was collected and dried under vacuum. Dried lipids were further solubilised in a CHCl_3_/MeOH/H_2_O (60/40/4.5) mixture before quantitative analysis of phospholipids by liquid chromatography coupled with tandem mass spectrometry (LC-MS/MS). Phospholipids were analysed on a Zorbax RX-Sil C18 100 × 2.1 mm, 1.8-μm column (Agilent Technologies) connected to a 1200 HPLC (Agilent Technologies) using a binary gradient of solvent A (10 mM ammonium acetate and 1 mM acetylacetone in water/methanol 60:40) and B (10 mM ammonium acetate and 1 mM acetylacetone in isopropanol/methanol 90:10). Positive electrospray ionisation mass spectrometry (ESI-MS) was performed on a QQQ 6460 mass spectrometer (Agilent Technologies) in the precursor ion mode (product ion m/z 184). Precursor ions are listed in Supplementary Table 1.

Chromatograms were analysed with the Agilent MassHunter Workstation software. Concentrations of PC and LPC species were determined from the ratio of the peak area of a given species to the peak area of the internal standard. Concentrations were expressed in pmoles per microgram of proteins (pmoles/µg).

### Triglyceride quantification

Cells were washed with ice-cold PBS and the lipids were extracted by an overnight incubation with isopropanol. After an additional wash with isopropanol, lipid extracts were sequentially washed with toluene and chloroform, drying the samples between each solvent step. The lipids were subsequently dissolved with 1% Triton X-100 in chloroform before drying the solvent. Finally, the lipid-Triton mixtures were dissolved in water at 55 °C for 1 h before being cooled to room temperature. Triglyceride levels were measured enzymatically with a commercially available kit (Diasys, Holzheim, Germany). For protein measurements, a 0.1 M NaOH solution containing 1% SDS was added to the wells of the culture plates after lipid extraction and the plates were incubated for 2 h at room temperature. After an additional wash with 0.1 M NaOH/1% SDS, the samples were sonicated before protein measurement using a BCA assay. Cellular TG content was finally expressed as milligrams or micrograms of TG per milligram of total proteins.

### Lipid droplet isolation

Confluent monolayers of cells were collected and disrupted by homogenisation in TNE buffer (20 mM Tris-HCl, 130 mM NaCl, 5 mM EDTA; pH 8) using a Dounce type glass-Teflon homogeniser. After protein quantification, lysates containing equal proportions of proteins from each cell line were then mixed with TNE buffer containing 1 M sucrose (1:1 ratio) and centrifuged 10 min at 1000 × *g* at 4 °C. The post-nuclear supernatant (PNS) was transferred to a 15-mL ultracentrifuge tube (Beckman Coulter). Then 3.0 mL of sucrose 0.25 M, 3.0 mL of sucrose 0.125 M and TNE buffer were layered sequentially. The step-wise gradient was centrifuged at 28,000 rpm, at 4 °C, for 2 h using the SW41 rotor (Beckman Coulter). The top of the gradient corresponding to the LD fraction was collected with a Pasteur pipette. The middle, corresponding to the cytosolic fraction, and the pellet, corresponding to the total membrane fraction, were collected. The LD fraction was washed with TNE pH 11. LD and cytosolic fraction proteins were extracted and precipitated with MeOH/CHCl_3_. Each fraction was then mixed with RIPA buffer for protein quantification.

### Transmission electron microscopy

Cells were fixed with 4% glutaraldehyde, 2.5% paraformaldehyde in 0.1 M Sorensen phosphate buffer, pH 7.4, 1 h at room temperature. After subsequent buffer washes, the cells were included in agarose 3% and postfixed in 2.0% osmium tetroxide for 1 h at room temperature and then washed again in buffer followed by distilled water. After dehydration through graded ethanol series, the tissue was infiltrated and embedded in EMbed-812 resin (Electron Microscopy Sciences). Thin sections were examined with a Hitachi 75000 electron microscope.

### Immunohistochemistry

Four-micrometre slices were cut from formalin-fixed paraffin-embedded samples. Wax was automatically removed using a Leica Autostainer (Nussloch) and then stained with CD8 antibody or LPCAT2 antibody (Supplementary Table 4) at 1:200 using a Ventana Benchmark (Tucson). Absolute quantification of CD8-positive cells was carried out with Tissue Studio 4.0 software (Definiens). Briefly, after numeration using the Nanozoomer 2.0 HT and NDP scan software (Hamamatsu), the slides were processed and analysed in tumour areas manually defined by the pathologist for each sample. Paracancerous sections of human thyroid samples were used as a positive control for LPCAT2 expression and staining (www.proteinatlas.org/ENSG00000087253-LPCAT2/tissue). Of the 79 hepatic metastasis samples from CRC patients, a double-blinded scoring of staining intensity was performed to discriminate between samples with high and low LPCAT2 expression.

### Nile red/ER tracker staining

Live cells seeded on cover glasses were incubated with Nile red (Molecular Probes) at 1:2000 in PBS for 15 min or with 1 μM ER Tracker (Molecular Probes) in DMEM for 30 min at 37 °C and then fixed in 4% PFA for 10 min at 4 °C. The slides were mounted in ProLong Gold Antifade with DAPI (Molecular Probes) before imaging. Images were acquired with an Axio Imager M2 (Zeiss) coupled with an Apotome.2 (×63 or ×40 objective) and analysed with ImageJ and Icy software programmes.

### Immunofluorescence staining

For double staining of LPCAT2/CRT and Bodipy 493/503, cells were fixed in 4% PFA for 10 min at 4 °C and permeabilised with PBS 1% BSA 0.2% saponin for 30 min. Cells were then incubated with LPCAT2 or CRT antibody (Supplementary Table 4) at 1:250 overnight at 4 °C. Secondary Alexa Fluor 568 goat anti-mouse or anti-rabbit (Molecular Probes) was used at 1:1000 with BODIPY 493/503 at 1:2000 for 45 min at room temperature. For PDI staining, cells were fixed and permeabilised with glacial methanol for 10 min on ice. After 10 min of saturation at room temperature with saturation buffer (PBS 1×, 1% BSA and 0.2% Triton X-100), cells were then incubated with PDI antibody (Supplementary Table 4) in saturation buffer for 1 h at 4 °C. After intracellular staining, cells were incubated with a mix of goat anti-mouse Alexa Fluor 488 antibody (1:1000) in saturation buffer for 45 min at room temperature. Isotype-matched IgG antibodies were used as a control. Slides were mounted in ProLong Gold Antifade with DAPI before imaging. Images were analysed with Image J and Icy software.

### BODIPY 493/503

Cells were incubated with BODIPY 493/503 (Molecular Probes) at 1:2000 and NucBlue® (Molecular Probes) in PBS for 15 min at room temperature. Cells were fixed in 4% PFA for 10 min at 4 °C, washed once in PBS and analysed by flow cytometry on a BD LSR-II cytometer equipped with BD FACSDiva software (BD Biosciences) and the data were analysed using FlowJo software (Tree Star, Ashland, OR, USA). Gating strategies are presented in Supplementary Fig. 10.

### Immunostaining analysis by flow cytometry

All flow cytometry experiments were conducted on a BD LSR-II cytometer equipped with BD FACSDiva software (BD Biosciences) and data were analysed using FlowJo software (Tree Star, Ashland, OR, USA). Gating strategies are presented in Supplementary Fig. 10.

For Ki67 staining cells were fixed and permeabilised with the glacial methanol for 10 min on ice. After 10 min of saturation at room temperature with saturation buffer (PBS 1×, 1% BSA and 0.2% Triton X-100), cells were then incubated with Ki67 antibody (Supplementary Table 4) in saturation buffer for 1 h at 4 °C. After intracellular staining, cells were incubated with a mix of goat anti-mouse Alexa Fluor 488 antibody (1:1000) and NucBlue® (2 drops/mL of solution) in saturation buffer for 45 min at room temperature. For CRT staining, cells were incubated 1 h at 4 °C with CRT antibody (Supplementary Table 4) for cell surface staining and then incubated for 45 min at room temperature with a solution of anti-mouse Alexa Fluor 488 (1:1000) and NucBlue^®^ (2 drops/mL of solution) (Thermo Fisher Scientific). For PLIN2/CRT double staining, after incubation with CRT antibody and extensive washings, cells were fixed and permeabilised with intracellular fixation and permeabilisation buffer (eBiosciences). Then cells were incubated with PLIN2 antibody (Supplementary Table 4) for 1 h at 4 °C. After intracellular staining, cells were incubated with a mix of goat anti-rabbit-Alexa Fluor 568, goat anti-mouse Alexa Fluor 488 (1:1000 each) and NucBlue® for 45 min at room temperature. Isotype-matched IgG antibodies were used as a control and fluorescence intensity of stained cells was gated on live cells.

### Isolation and analysis of tumour-infiltrating CD8^+^ T cells

Eight-week-old female balb/c mice bearing CT26-lpcat2 or CT26-Ctl tumours were subdivided into two groups and intraperitoneally injected with vehicle or FOX. One week after injection, allograft tumours were dissociated in RPMI with the gentleMACS^TM^ Dissociator (Myltenyi Biotec) according to the manufacturer’s protocol. Lysates were centrifuged and cells were stained with an antibody cocktail containing anti-CD45, CD3, CD8, PD-1 and Tim-3 antibodies (Supplementary Table 4) for 1 h at 4 °C. After surface staining, 100 µL of red blood cell lysis solution (BD Biosciences) was added for 10 min. Cells were centrifuged (400 × *g*, 5 min), resuspended in flow cytometry buffer (eBiosciences), transferred into Trucount^TM^ Tubes (BD Biosciences) and then analysed on a BD LSR-II cytometer. The gating strategy is described in Supplementary Fig. 10.

### ELISA

After tumour dissociation supernatants were assayed by ELISA for mouse IFN-γ secretion (BD Biosciences), according to the manufacturer’s protocol.

### Western blot analysis

Cells were lysed in RIPA buffer (50 mM Tris, 150 mM NaCl, 0.5% NaDeoxycholate, 1% NP40, 2 mM EDTA, 50 mM NaF, 100 µM PMSF; pH 8) containing complete ultra-protease/phosphatase inhibitor (Roche). Proteins were resolved by SDS-PAGE and transferred to nitrocellulose membranes (Amersham). Blots were then saturated in 5% milk (1 h at room temperature) before overnight incubation at 4 °C with specific primary antibodies (Supplementary Table 4). All primary antibodies were diluted at 1:1000 in 5% w/v non-fat milk or 5% BSA. Primary antibodies were detected using horseradish peroxidase (HRP)-conjugated appropriate secondary antibodies (Cell Signaling Technologies) followed by exposure to ECL (Santa Cruz Biotechnology). A signal was acquired with a ChemiDoc^TM^ XRS + imaging system (Biorad) and blots were analysed with Image Lab^TM^ software 5.1.2 (Biorad, France). Uncropped blots of the main figures are presented in Supplementary Fig. 11.

### Statistical analysis

Statistical analysis was conducted using Prism software 6 (GraphPad Software). For the analysis of the experimental data, continuous data were compared using the Mann–Whitney *U* test, multiple Student *t* tests or two-way ANOVA as appropriate, after having checked data for normal distribution and variance homogeneity. All *p* values are two-tailed; *p* values <0.05 were considered significant (*p < 0.05, **p < 0.01 and ***p < 0.001). The data are represented as mean ± s.e.m. or the median with 10 and 90 percentiles. Animal experiments were not blinded. As regards clinical data, patient characteristics were examined using Mann–Whitney tests for continuous variables. All patients were followed-up until death or the end of data recording. Recurrence-free survival (RFS) was calculated from the date of diagnosis until the date of relapse (local or metastatic). Alive or deceased patients without relapse were censored at the last follow-up. RFS probabilities were estimated by the Kaplan–Meier method and were compared with the log-rank test. Analyses were performed using the MedCalc statistical software package.

### Data availability

The authors declare that all the data supporting the findings of this study are available within the article and its supplementary information files or from the corresponding authors upon reasonable request.

## Electronic supplementary material

Supplementary Information
