# Supplementary material for: Lysophosphatidylcholine acyltransferase 2-mediated lipid droplet production supports colorectal cancer chemoresistance
Source: Nat Commun. 2018 Jan 22;9:322. doi: 10.1038/s41467-017-02732-5 (PMC5778070; doi:10.1038/s41467-017-02732-5)
Supplement: Supplementary file 1 — Supplementary Information [file 41467_2017_2732_MOESM1_ESM.pdf]

a

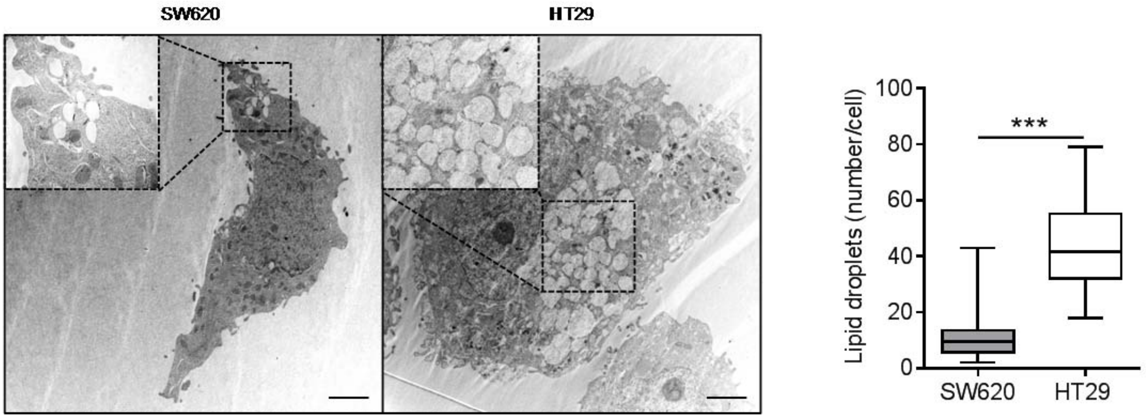

b

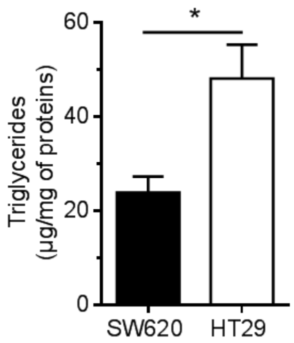

Supplementary Figure 1. Lipid droplet accumulation is preponderant in high-LPCAT2 HT29 cells. a) Electron microscopy was performed on SW620 and HT29 cells after 48 h of seeding. Left panel, representative images of TEM (scale bar = 2 µm, scale bar of insert = 500 nm). Right panel, LD quantification was performed by counting total lipid bodies per cell. A total of 50 cells per cell line were counted. Data are expressed as LD number/cell with whiskers denoting the 1st and 99th percentiles. *P*-values were determined by Mann-Whitney U-test. *** p<0.001. **b)** Triglyceride quantification in SW620 and HT29 cells after 48 h of seeding. Data are expressed as µg of triglycerides per mg of proteins and are means ± s.e.m. of three independent experiments. *P*-values were determined by Mann-Whitney U-test. * p<0.05.

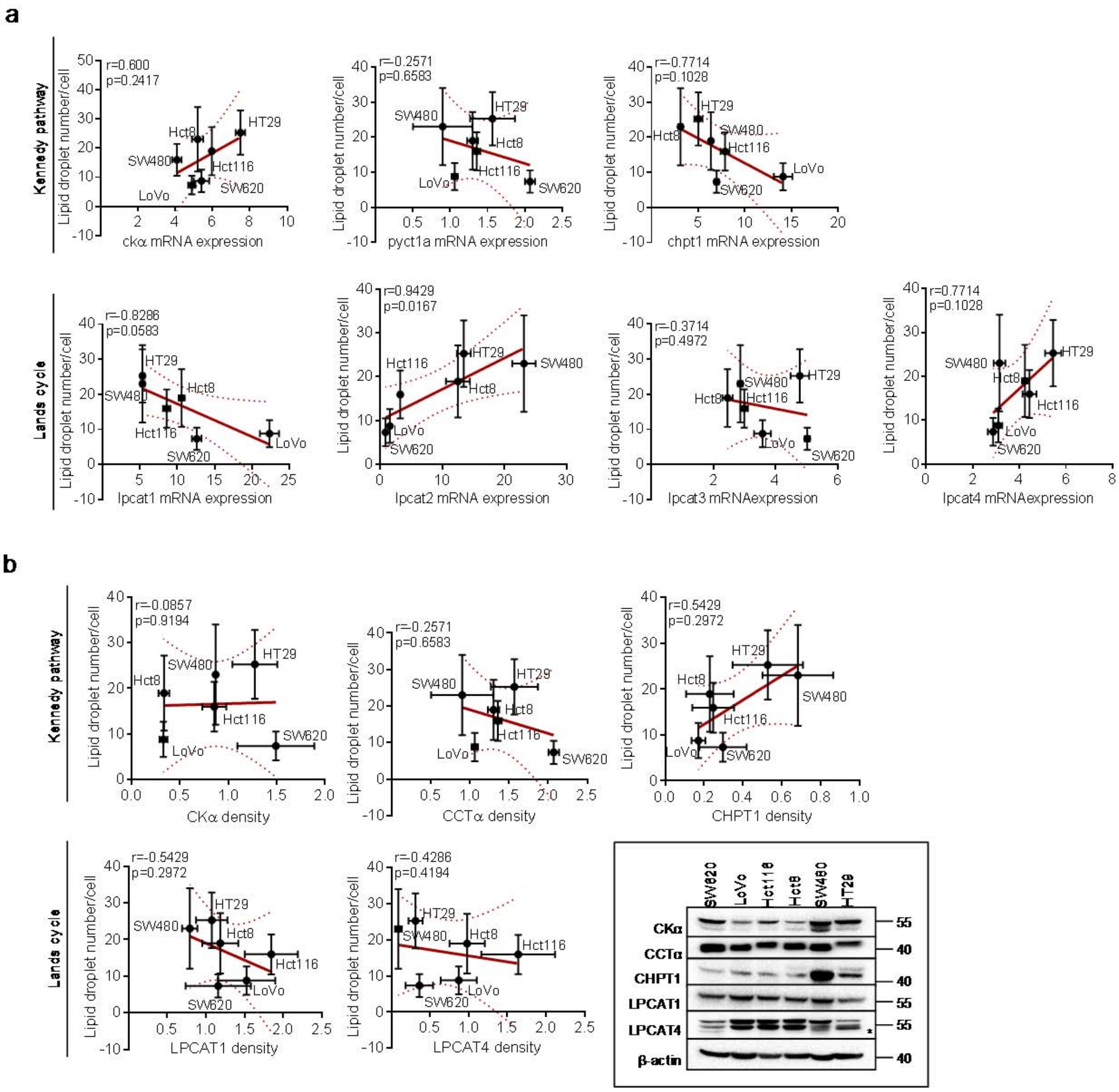

Supplementary Figure 2. Only LPCAT2 expression correlates with LD density in CRC cell lines a) Relative mRNA expression levels of *Ckα*, *Chpt1*, *Cctα*, *Lpcat 1*, 2, 3 and 4 were assessed by RT-qPCR in SW620, LoVo, Hct116, Hct8, SW480 and HT29 cell lysates after 48 h of seeding. Spearman's correlation between each mRNA expression level and LD content was determined from three independent experiments. **b)** Spearman's correlation between each protein expression level and LD content was determined from three independent experiments. Insert: Representative western-blot of CKα, CHPT1, CCTα, LPCAT 1, 2 and 4 proteins expression performed on SW620, LoVo, Hct116, Hct8, SW480 and HT29 cell lysates after 48 h of seeding.

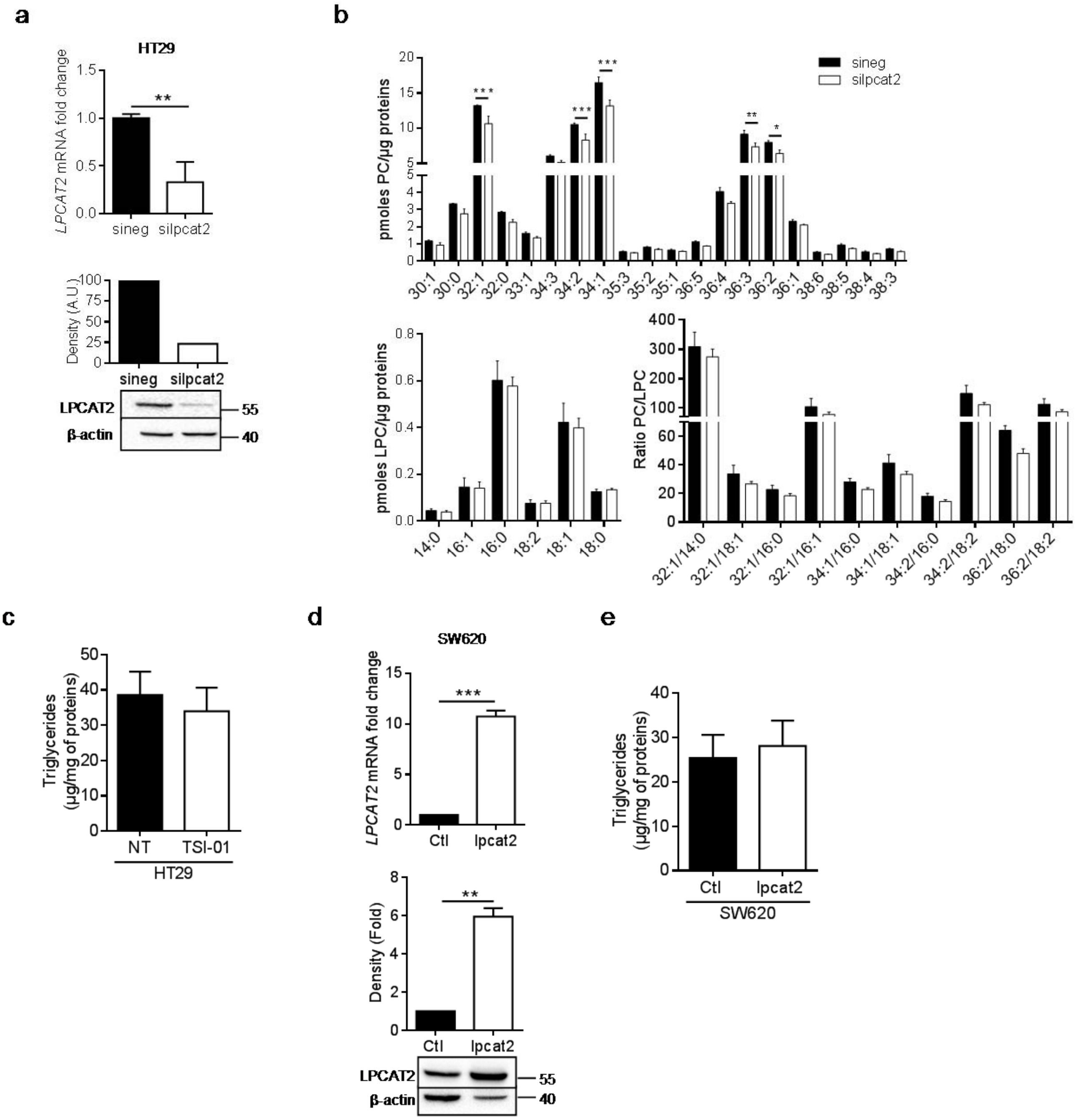

Supplementary Figure 3. Phosphatidylcholine metabolism is preponderant in high-LPCAT2 cells. a) RT-qPCR mRNA expression analysis (upper panel) and western blots (lower panel) showing the knockdown efficiency of siRNA against *Lpcat2* (silpcat2) in HT29 cells 72 h after transfection. Scrambled siRNA (sineg) was used as control. Data represent three independent experiments. *P*-value was determined by student's t-test. **p<0.01. Error bars denote s.e.m. **b)** Molecular species of PC and LPC total pools described in figure 1d, in HT29 cells transfected with siRNA targeting *Lpcat2* (silpcat2) *vs* scrambled siRNA (sineg), were assessed by positive ESI-MS/MS. Data are mean ± s.e.m. of three independent experiments. *P*-values were determined by student's t-test. * p<0.05, ** p<0.01, *** p<0.001. **c)** Triglyceride quantification in HT29 cells after 48 h of treatment with vehicle (DMSO, NT) or TSI-01 (10 µM). *P*-value was determined by Mann-Whitney U-test. Error bars denote s.e.m. **d)** RT-qPCR (upper panel) and Western-blot (lower panel) were performed on SW620 cells stably mock-infected (SW620-Ctl) or SW620 cells stably infected with *Lpcat2*-PCMV6 vector (SW620-lpcat2) after 48 h of seeding to assess LPCAT2 overexpression efficiency. Data represent three independent experiments. *P*-value was determined by Mann-Whitney U-test. ** p<0.01. Error bars denote s.e.m. **e)** Triglyceride quantification in SW620-Ctl *vs* SW620 overexpressing LPCAT2 (SW620-lpcat2) after 48 h of seeding. Data are expressed as µg of triglycerides per mg of proteins and are mean ± s.e.m. of three independent experiments. *P*-values were determined by Mann-Whitney U-test.

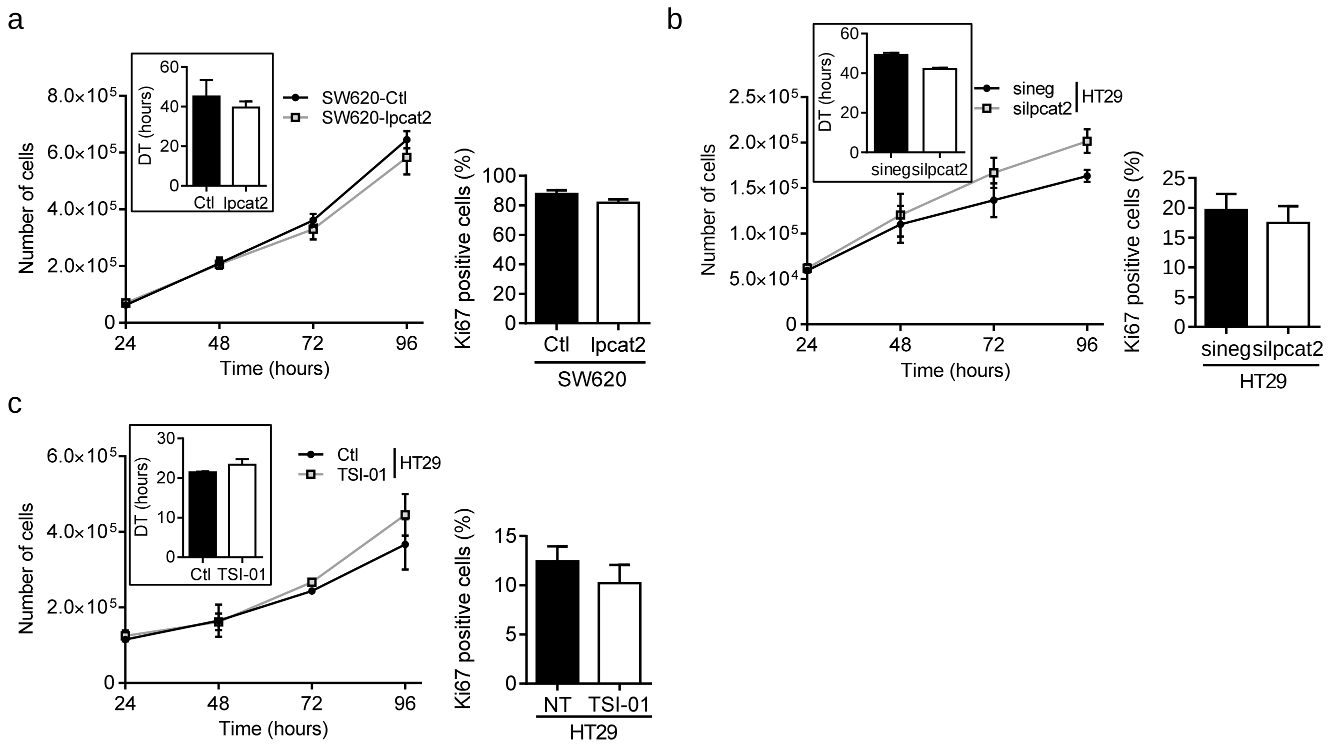

Supplementary Figure 4. Cell proliferation rate is independent of LPCAT2 expression. (a-c) Impact of LPCAT2 on growth curves (left panels) and proliferation rates assessed by Ki67 staining (right panels) in: **a)** SW620-ctl *vs* SW620-lpcat2; **b)** HT29 cells transiently transfected with sineg *vs* silpcat2; **c)** HT29 cells treated with vehicle *vs* TSI-01. Inserts represent doubling times in the various experimental conditions. Growth curves p-values were determined by a two-way ANOVA with Bonferroni correction. For DT and percentages of Ki67 positive cells, p-values were determined by Mann-Whitney U-test. Data are mean ± s.e.m. of three independent experiments.

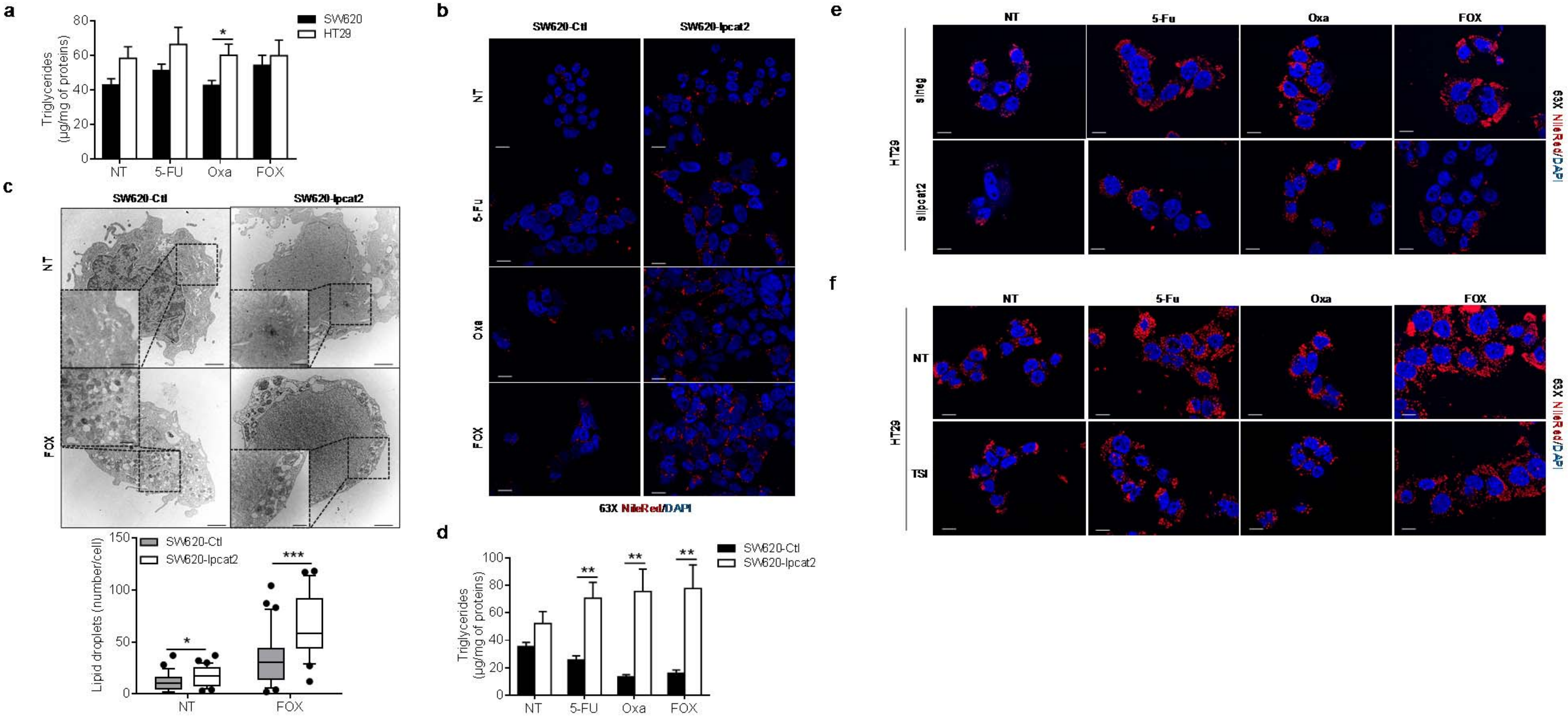

Supplementary Figure 5. LPCAT2 supports LD production in CRC cell lines under chemotherapeutic treatment. a) Triglyceride quantification in SW620 and HT29 cells treated for 48 h with vehicle (DMSO, NT), 5-Fu (10 µM), Oxa (10 µM) or FOX (5-Fu + Oxa, 10 µM each). Data are expressed as µg of triglycerides per mg of proteins and are mean ± s.e.m. of three independent experiments. *P*-values were determined by multiple student's t-test. * $p<0.05$. **b)** Representative confocal microscopy images (63X magnification) of Nile red staining after 48 h of treatment with vehicle (DMSO, NT), 5-Fu (10 µM), Oxa (10 µM) or FOX (5-Fu + Oxa, 10 µM each) in SW620- Ctl *vs* SW620 overexpressing LPCAT2 (SW620-lpcat2). **c)** Electron microscopy was performed on SW620 and HT29 cells after 48 h of treatment with vehicle (DMSO, NT) or FOX (5-Fu + Oxa, 10 µM each). Upper panel, representative images of TEM (scale bar = 2 µm, scale bar of insert = 500 nm). Lower panel, LD quantification was performed by counting total lipid bodies per cell. A total of 50 cells per cell line were counted. *P*-values were determined by multiple student's t-test. * $p<0.05$, *** $p<0.001$. **d)** Triglyceride quantification in SW620- Ctl *vs* SW620-lpcat2 treated as in a). *P*-values were determined by multiple student's t-test. ** $p<0.01$. Error bars denote s.e.m. **e-f)** Representative confocal microscopy images (63X magnification) of Nile red staining in **e**) HT29 cells transiently transfected with scrambled siRNA (sineg) *vs* *Lpcat2* siRNA (silpcat2) and treated as in b), and in **f)** vehicle (DMSO) *vs* TSI-01 (10µM) cotreatment.

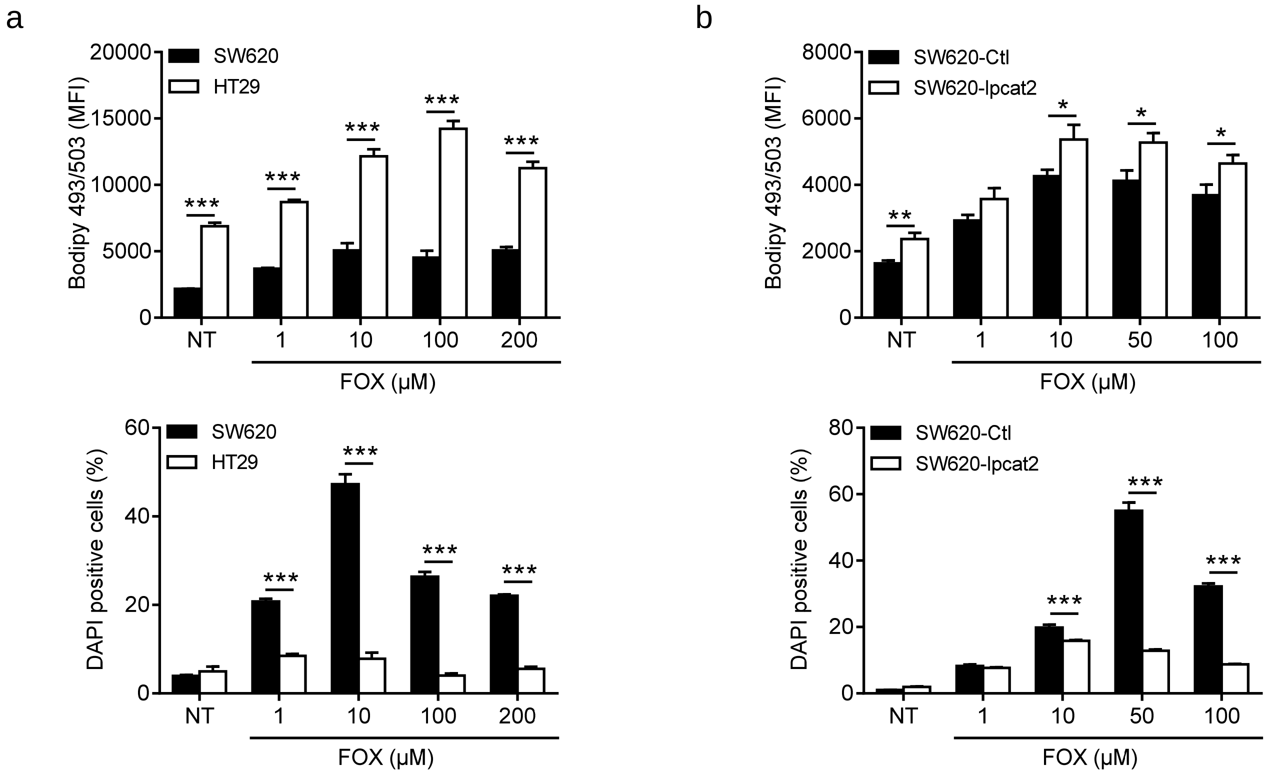

Supplementary Figure 6. Lipid droplet accumulation is dependent on chemotherapy concentration. a) SW620 *vs* HT29 cells **b)** SW620- Ctl *vs* SW620 overexpressing LPCAT2 (SW620-lpcat2) were stained with Bodipy 493/503 and analyzed by flow cytometry after 48 h of treatment with increased concentrations of FOX (5-Fu + Oxa). Bodipy 493/503 Median of Fluorescence Intensity (MFI) was used to assess LD content (upper panel). Percentage of DAPI positive cells was analyzed for each drug concentration in three independent experiments. Multiple student's t-test was used to compare cell lines for each FOX concentration. Data are mean ± s.e.m. of three independent experiments. * $p<0.05$, ** $p<0.01$, *** $p<0.001$.

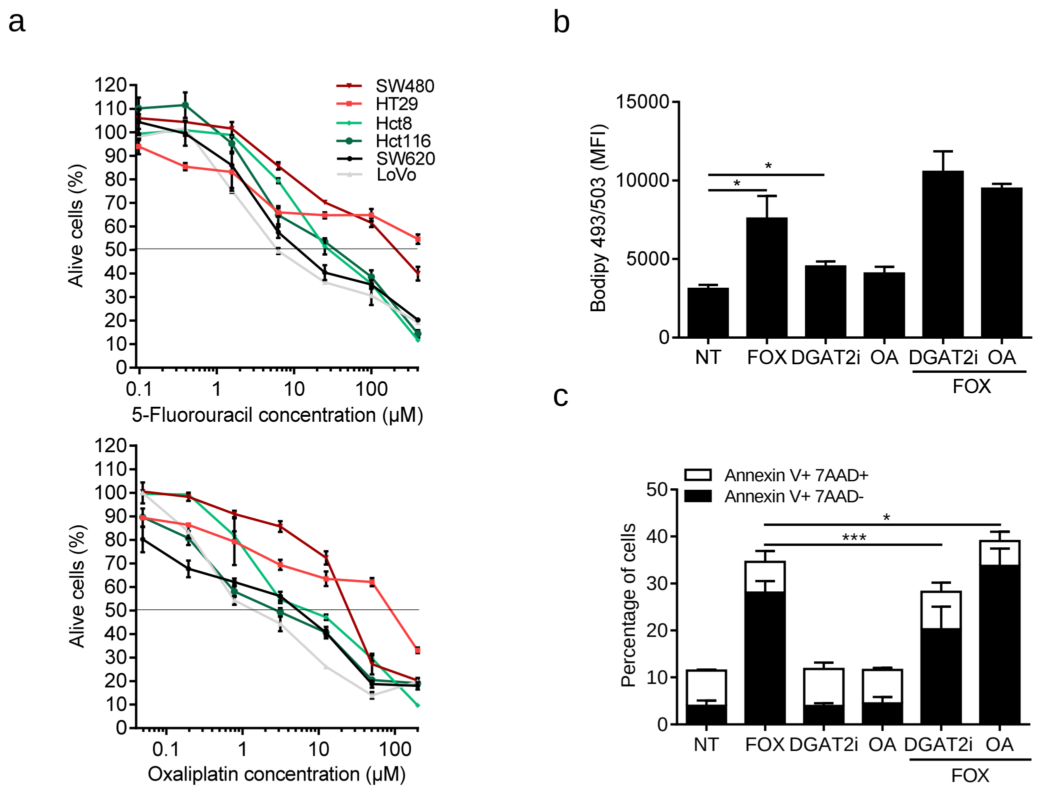

Supplementary Figure 7. DGAT2 and oleic acid are not involved in LD-dependent resistance to chemotherapy. **a)** Cell viability of SW620, HT29, LoVo, Hct116, SW480 and Hct8 was determined by crystal violet staining, after 48 h of treatment with concentration ranges of 5-Fu (starting concentration 400 µM, 1:4 serial dilutions) and Oxa (starting concentration 200 µM, 1:4 serial dilutions). The inhibitory concentration 50% (IC_{50}) for each cell line was determined by a 4-parameter nonlinear regression. Error bars denote s.e.m. **b)** HT29 cells were stained with Bodipy 493/503 and analyzed by flow cytometry after 48 h of treatment with vehicle (DMSO, NT), FOX (5-Fu + Oxa, 10 µM each), and DGAT2 inhibitor (DGAT2i, 10 µM), oleic acid (OA, 10 µM) alone or in combination with FOX therapy. Bodipy 493/503 Median of Fluorescence Intensity (MFI) was used to assess LD content in three independent experiments. *P*-values were determined by a two-way ANOVA with Bonferroni correction. * $p<0.05$. Error bars denote s.e.m. **c)** HT29 cells were treated as described in b) and stained with Annexin V/7AAD. *P*-values were determined by a two-way ANOVA with Bonferroni correction. * $p<0.05$, *** $p<0.001$. Data are mean ± s.e.m. of three independent experiments.

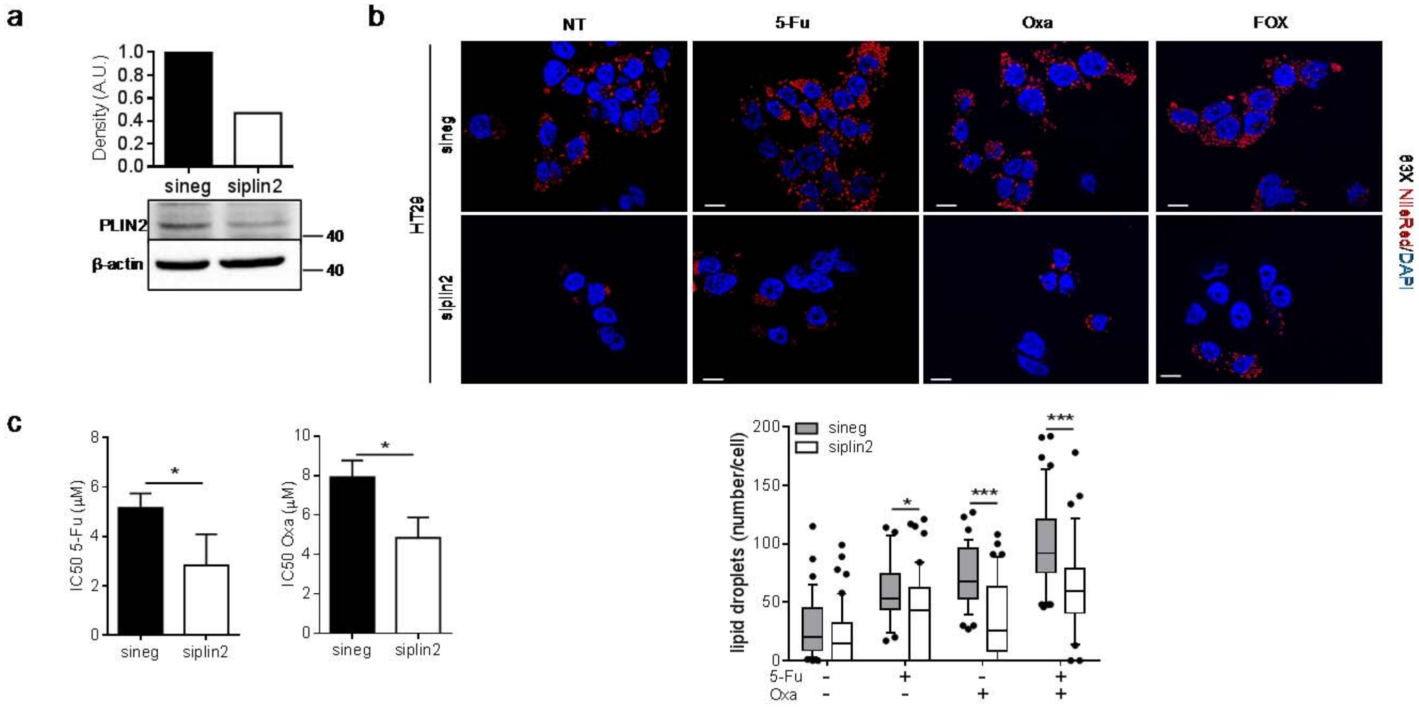

Supplementary Figure 8. PLIN2 is involved in LD-dependent resistance to chemotherapy a) Western-blot analysis on HT29 cells transfected during 72h with scrambled siRNA (sineg, 10 nM) or with siRNA against *Plin2* (siplin2, 10 nM). **b)** HT29 cells transfected as in a) were stained with Nile red, 48 h after vehicle (DMSO, NT), 5-Fu (10 µM), Oxa (10 µM) or FOX (5-Fu + Oxa, 10 µM each) treatments. Representative images of Nile red staining from three independent experiments are shown (Left panel). LD quantification was performed by counting red lipid bodies on merged pictures (300 cells per condition) (Right panel). Data are expressed as LD number/cell with whiskers denoting the 1st and 99th percentiles. *P*-values were determined by a two-way ANOVA with Bonferroni correction. *** $p<0.001$. **c)** Cell viability was determined by crystal violet staining, after 48 h of treatment with concentration ranges of 5-Fu (starting concentration 400 µM, 1:4 serial dilutions) and Oxa (starting concentration 200 µM, 1:4 serial dilutions) either in HT29 cells transfected with scrambled siRNA (sineg) or with siRNA against *Plin2*. The inhibitory concentrations 50% (IC_{50}) were determined by a 4-parameter nonlinear regression. *P*-values were determined by student's t-test. * $p<0.05$. Data are mean ± s.e.m. of three independent experiments.

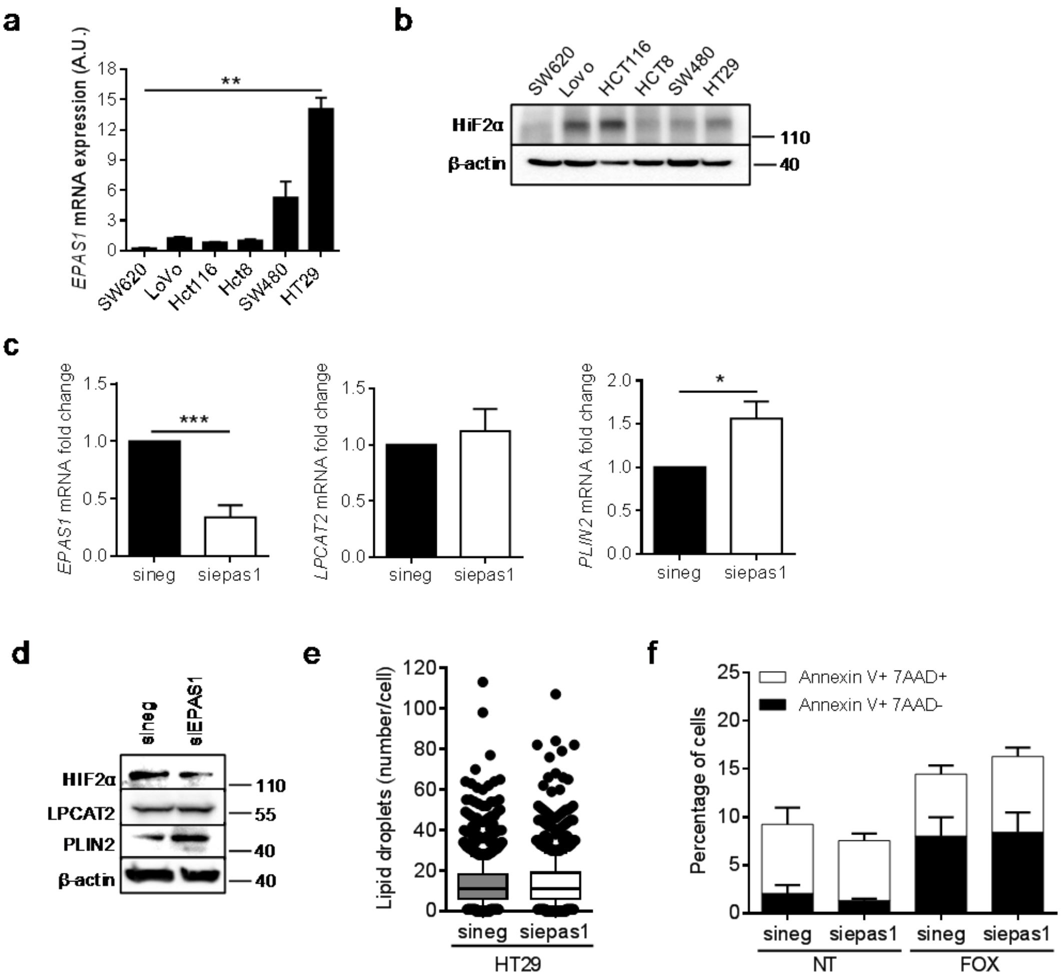

Supplementary Figure 9. PLIN2 involvement in LD-dependent resistance to chemotherapy is an HIF2α-independent mechanism. (a-b) Analysis **o**n SW620, HT29, LoVo, Hct116, SW480 and Hct8 after 48 h of seeding. **a)** RT-qPCR mRNA expression of *Epas1.* **b)** Western-blot analysis of HIF2-α protein expression. A representative blot from three independent experiments is shown. **c)** RT-qPCR mRNA expression analysis showing knockdown efficiency of siRNA against *Epas1* (siepas1), *Plin2* and *Lpcat2* gene expression in HT29 cells after 48 h of transfection. Scrambled siRNA (sineg) was used as control. Data represent three independent experiments. *P*-value was determined by student's t-test. * p<0.05, *** p<0.001. **d)** Western-blot analysis of HIF2α, PLIN2 and LPCAT2 proteins after 72 h of transfection with siRNA against *Epas1* (siepas1) or scrambled siRNA (sineg). **e)** HT29 cells were stained with nile red after 72 h of transfection with siRNA against *Epas1* (siepas1) or scrambled siRNA (sineg). LD quantification was performed by counting red lipid bodies on merged pictures (300 cells per condition) with Image J software. Data are expressed as LD number/cell with whiskers denoting the 1st and 99th percentiles. *P*-values were determined by a two-way ANOVA with Bonferroni correction. **f)** Annexin V/7AAD staining on HT29 cells transiently transfected with sineg *vs* siepas1, untreated (NT) or treated with FOX for 48 h (FOX).

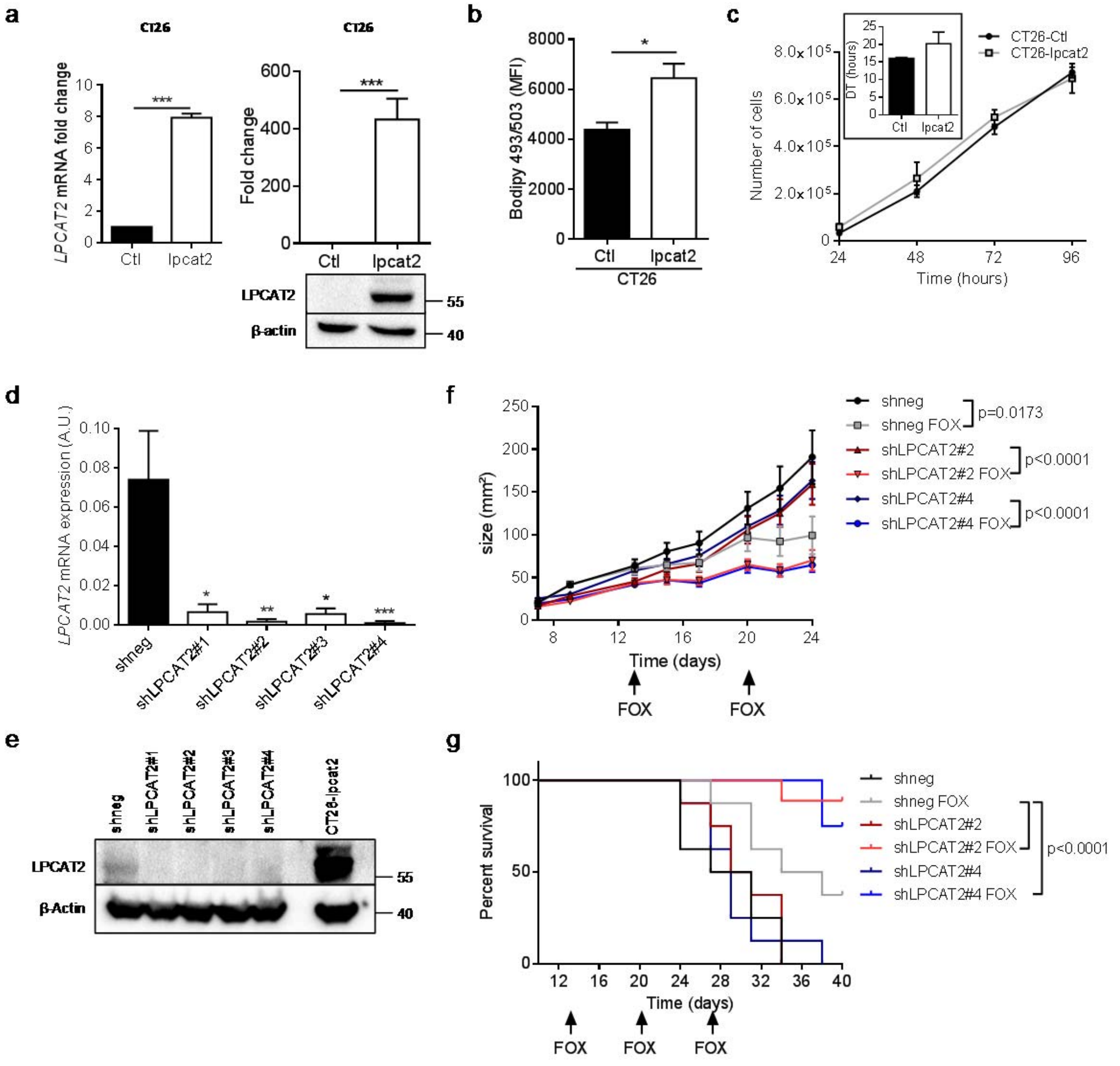

Supplementary Figure 10. LPCAT2 also supports resistance in murine colorectal cancer model. a) RT-qPCR (left panel) and Western-blot (right panel) were performed on CT26 cells stably mock-infected (empty-PCMV6 vector-containing viral particles, CT26-Ctl) or CT26 cells stably infected with *Lpcat2*-PCMV6 vector-containing viral particles (CT26-lpcat2) after 48 h of seeding to assess LPCAT2 overexpression efficiency. Data represent three independent experiments. *P*-values were determined by student's t-test. *** p<0.001. Error bars denote SEM. **b)** LD content in CT26-Ctl *vs* CT26-lpcat2 was evaluated by flow cytometry after Bodipy 493/503 staining. *P*-value was determined by student's t-test. * p<0.05. Error bars denote s.e.m. **c)** Growth curves and doubling time (insert) of CT26-Ctl *vs* CT26-lpcat2. Growth curves p-values were determined by a two-way ANOVA with Bonferroni correction and for DT by student's t-test. Error bars denote s.e.m. **d)** RT-qPCR and **e)** Western-blot were performed on CT26 cells stably mock-infected (scrambled-shRNA vector-containing viral particles, shneg) or CT26 cells stably infected with four different *Lpcat2*-shRNA vector-containing viral particles (sh*Lpcat2*#1;2;3;4) after 48 h of seeding to assess LPCAT2 knockdown efficiency. Protein lysate of CT26 overexpressing LPCAT2 was used as positive control of LPCAT2 molecular weight and expression. Data represent three independent experiments. *P*-values were determined by student's t-test. *** p<0.001. Error bars denote s.e.m. **f)** 8-week-old female balb/c mice were split into 6 groups (N=10 per group). CT26-shneg and CT26-sh *Lpcat2*#2 or #4 tumor-bearing mice received once a week during three weeks i.p. FOX injections (5-Fu, 5 mg/kg + Oxa, 6 mg/kg). *P*-values were determined by a two-way ANOVA with Bonferroni correction. Tumor size in mm² over time is represented as median ± s.e.m. **g)** Kaplan-Meier cumulative survival plots for mice groups described in f), with p-values assessed by Log-rank test.

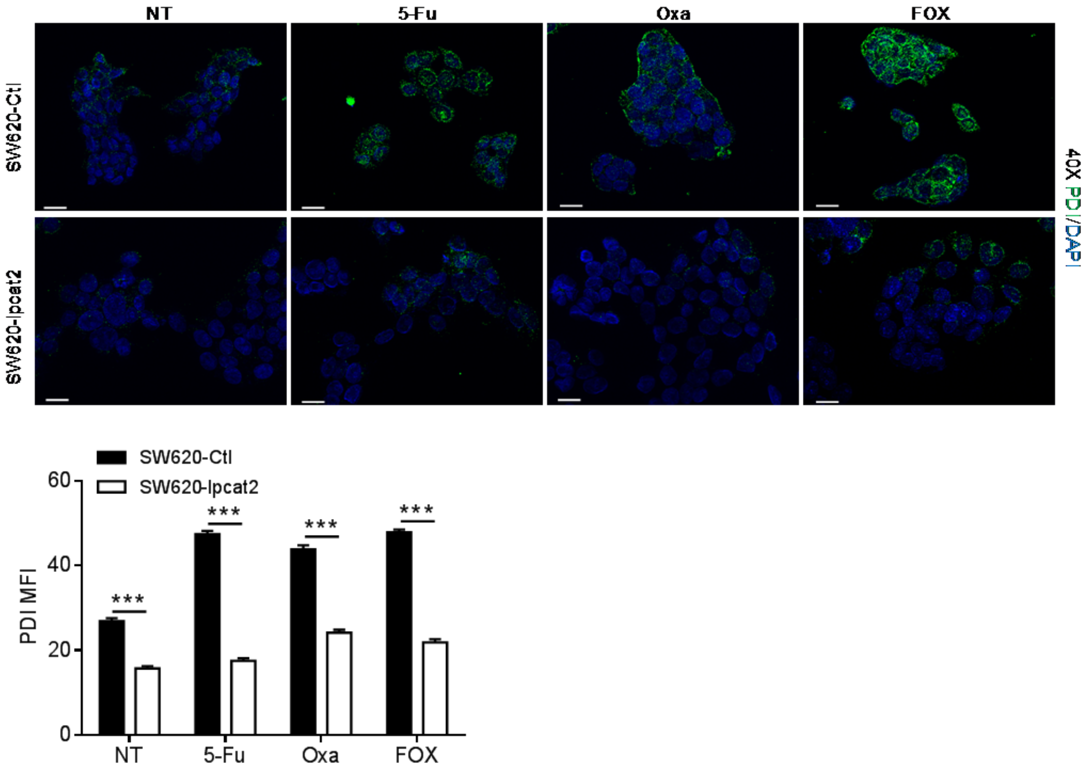

Supplementary Figure 11. LPCAT2 expression protects CRC cells against ER stress induction. SW620-Ctl and SW620-lpcat2 cells were stained with PDI antibody conjugated with goat anti-rabbit Alexa488 antibody 48 h after vehicle (DMSO, NT), 5-Fu (10 µM), Oxa (10 µM) or FOX (5-Fu + Oxa, 10 µM each) treatments. Representative images are shown (40X magnification, scale bar = 20 µm) (upper panel). PDI Median of Fluorescence Intensity (MFI) per cell was calculated from three independent experiments (300 cells per condition) (lower panel). *P*-values were determined by multiple student's t-test. *** $p<0.001$. Data are mean ± s.e.m. of three independent experiments.

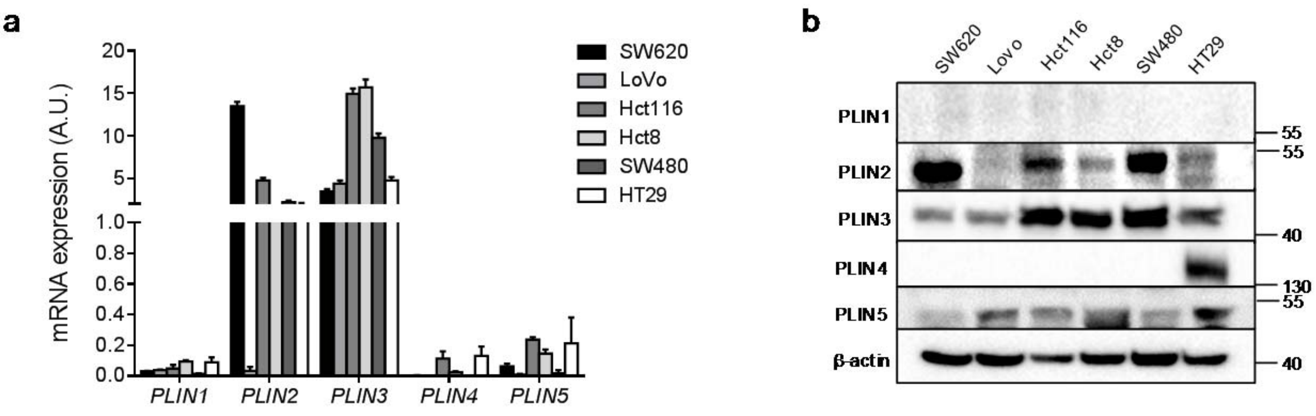

Supplementary Figure 12. Perilipin proteins are differentially expressed in CRC cells a) RT-qPCR and **b)** Western-blot analysis of PLIN1, PLIN2, PLIN3, PLIN4 and PLIN5 expression in SW620, LoVo, Hct116, Hct8, SW480 and HT29 cells lysates after 48 h of seeding. Data are mean ± s.e.m. of three independent experiments.

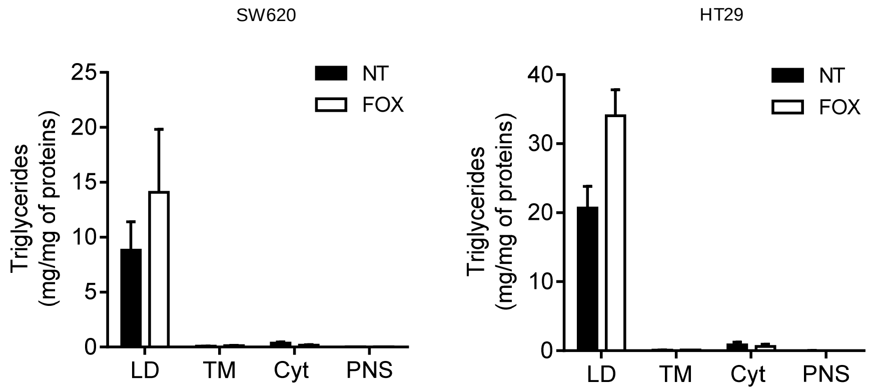

Supplementary Figure 13. Controls of fraction purity after LD isolation. Triglyceride quantification after LD isolation from confluent SW620 (Left panel) and HT29 (Right panel) cells treated or not with FOX for 48 h. Data are mean ± s.e.m. of three independent experiments and are expressed as mg of triglycerides per mg of proteins. Quantification was performed on LD, total membrane (TM), cytosol (cyt) and post-nuclear supernatant (PNS) fractions.

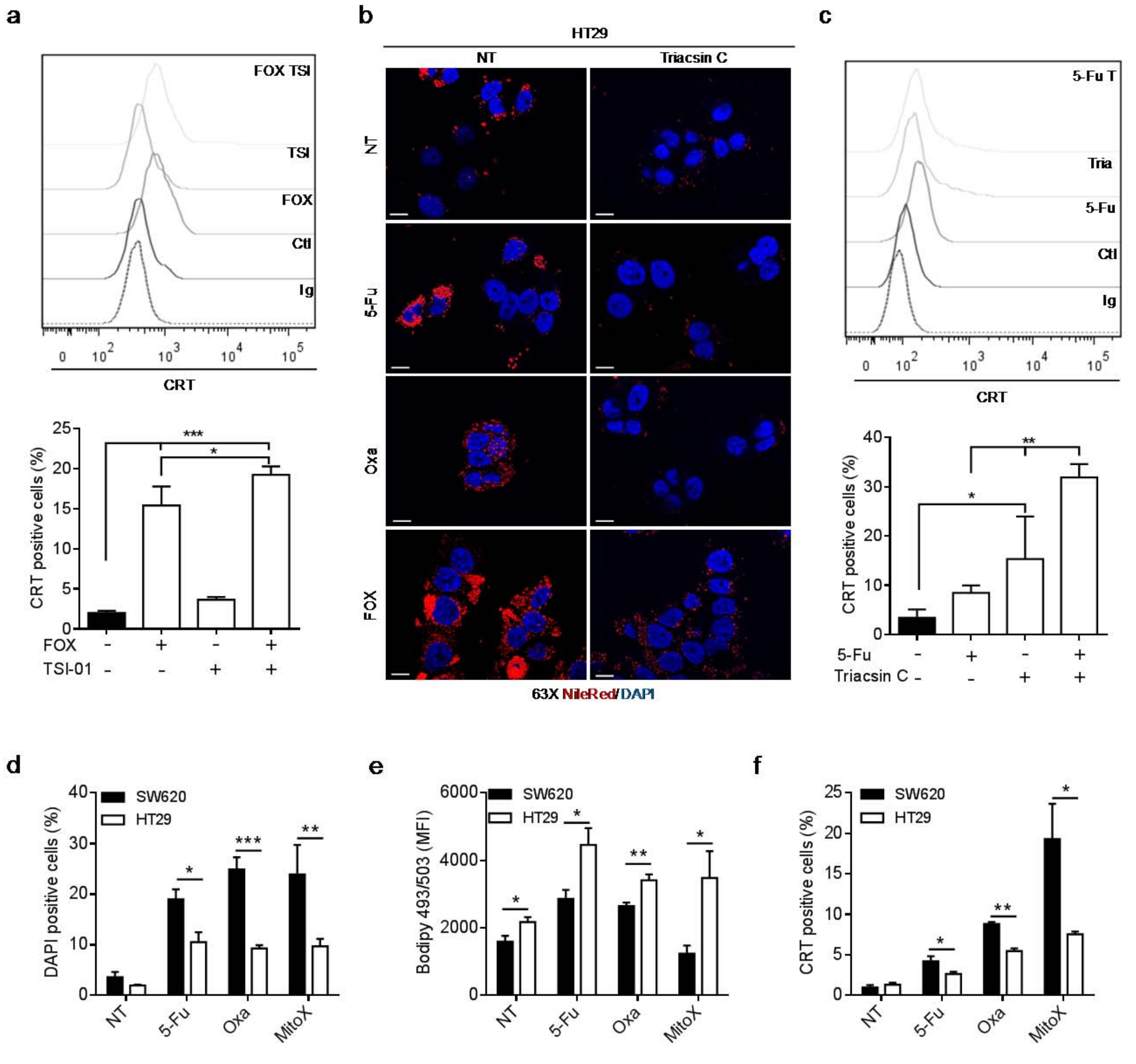

Supplementary Figure 14. CRT exposure is restored by LPCAT2 and LD inhibitors. a) Cell-surface CRT immunostaining was performed to assess CRT plasma membrane translocation in HT29 cells treated for 48 h with vehicle (DMSO, Ctl), TSI-01 or FOX (5-Fu + Oxa, 10 µM each) alone or with FOX + TSI-01 combination. **b)** Representative confocal microscopy images (63X magnification) of Nile red staining after 48 h of treatment with vehicle (DMSO, NT), 5-Fu (10 µM), Oxa (10 µM) or FOX (5-Fu + Oxa, 10 µM each) in the absence (NT) or presence of triacsin C (10 µM) in HT29 cells. **c)** Cell-surface CRT immunostaining was performed to assess CRT plasma membrane translocation in HT29 cells treated for 48 h with vehicle (DMSO, Ctl), 5-Fu (10 µM) or triacsin C (10 µM) alone or with 5-Fu + triacsin C. Percentages of CRT positive cells were determined for each treatment condition, as compared to the corresponding IgG control condition for each cell line. Data shown represent three independent experiments. *P*-values were determined by multiple student's t-test. * p<0.05, ** p<0.01, *** p<0.001. Error bars denote s.e.m. **d)** SW620 and HT29 cells were treated during 48 h with vehicle (DMSO, NT), 5-Fu (10 µM), Oxa (10 µM) or FOX (5-Fu + Oxa, 10 µM each), Mitoxantrone (MitoX, 1µM) and stained with DAPI. **e)** SW620 and HT29 cells treated as in d) were stained with Bodipy 493/503. Median of Bodipy fluorescence was calculated on DAPI negative cells. **f)** SW620 and HT29 cells treated as in d) were stained with CRT (FMC75) conjugated with goat anti-mouse Alexa488 antibody. Median of CRT fluorescence was calculated on DAPI negative cells. *P*-values were determined by multiple student's t-test. * p<0.05, ** p<0.01, *** p<0.001. Error bars denote s.e.m.

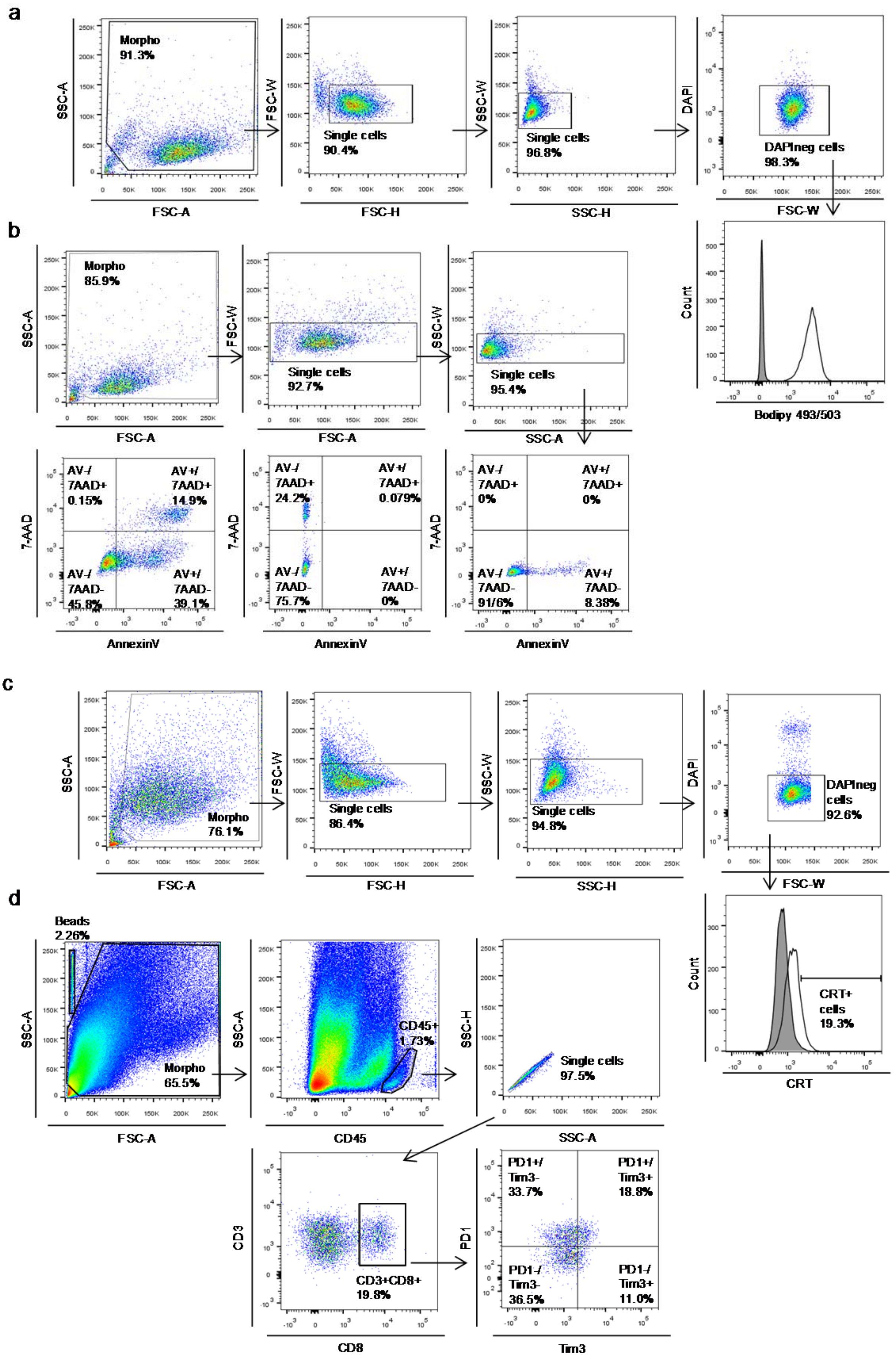

Supplementary Figure 15. Flow cytometry gating strategies. a) Gating strategy for Bodipy 493/503 staining. Grey histogram represents without Bodipy staining. **b)** Gating strategy for Annexin V/ 7AAD staining. **c)** Gating strategy for CRT staining. Grey histogram represents cells stained with Ig antibodies.**d)** Gating strategy for tumor-infiltrating CD8+ T cell population analysis.

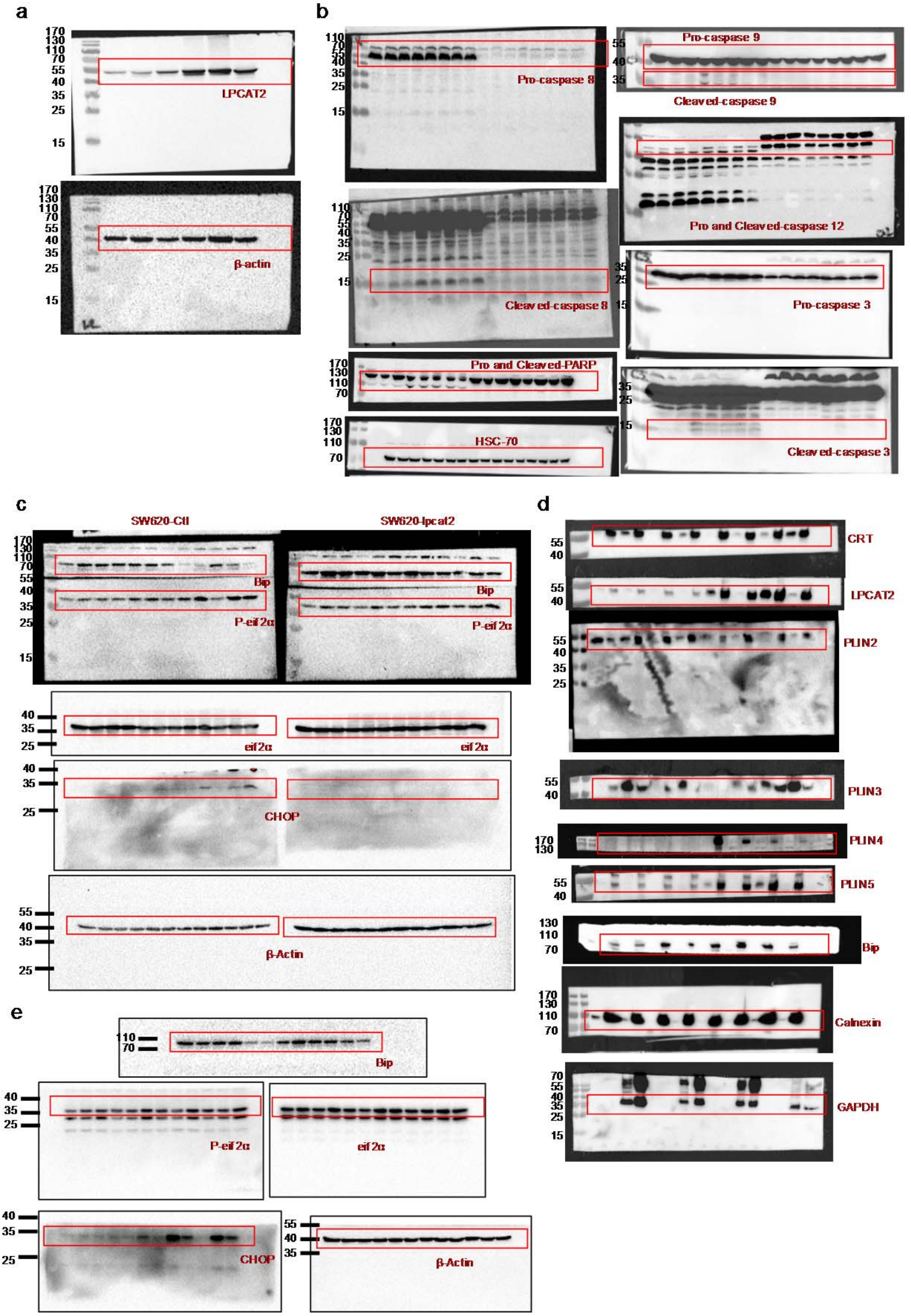

Supplementary Figure 16. Uncropped Western-blot images. The corresponding main figure numbers are shown. Red boxes highlight the cropped segment presented in main figures. Loading controls are provided. **a)** Fig. 1b **b)** Fig. 5a **c)** Fig. 5c **d)** Fig. 6b **e)** Fig. 7c.

Supplementary Table 1. Distribution of phosphatidylcholine (PC) and lysophosphatidylcholine (LPC) species in CRC cell lines.

m/z	Species	SW620 (pmoles/µg proteins)	HT29 (pmoles/µg proteins)	p-value	Summary
704.5	30:1 PC	0.72 +/- 0.027	1.04 +/- 0.056	0,3334	
706.5	30:0 PC	3.59 +/- 0.042	4.76 +/- 0.142	0,0006	***
730.5	32:2 PC	1.16 +/- 0.033	1.62 +/- 0.056	0,1642	
732.5	32:1 PC	11.03 +/- 0.216	12.05 +/- 0.267	0,0024	***
734.6	32:0 PC	2.14 +/- 0.100	2.95 +/- 0.071	0,0143	*
746.6	33:1 PC	1.48 +/- 0.060	1.97 +/- 0.020	0,1358	
754.5	34:4 PC	0.17 +/- 0.007	0.32 +/- 0.004	0,6389	
756.5	34:3 PC	1.63 +/- 0.029	2.49 +/- 0.060	0,0094	**
758.6	34:2 PC	10.42 +/- 0.236	11.8 +/- 0.347	< 0,0001	***
760.6	34:1 PC	21.20 +/- 1.564	23.11 +/- 0.849	< 0,0001	***
762.6	34:0 PC	0.74 +/- 0.038	0.96 +/- 0.027	0,4946	
770.6	35:3 PC	0.09 +/- 0.013	0.18 +/- 0.024	0,7751	
772.6	35:2 PC	0.69 +/- 0.024	0.85 +/- 0.031	0,6100	
774.5	35:1 PC	0.61 +/- 0.022	0.99 +/- 0.042	0,2381	
780.5	36:5 PC	0.25 +/- 0.013	0.49 +/- 0.009	0,4692	
782.6	36:4 PC	1.28 +/- 0.013	1.77 +/- 0.036	0,1332	
784.6	36:3 PC	3.13 +/- 0.109	4.2 +/- 0.080	0,0015	**
786.6	36:2 PC	12.2 +/- 0.720	12.24 +/- 0.296	0,9106	
788.6	36:1 PC	3.77 +/- 0.120	5.01 +/- 0.118	0,0002	**
806.6	38:6 PC	0.14 +/- 0.009	0.26 +/- 0.004	0,7134	
808.6	38:5 PC	0.31 +/- 0.016	0.55 +/- 0.013	0,4692	
810.6	38:4 PC	0.27 +/- 0.011	0.49 +/- 0.016	0,4882	
812.6	38:3 PC	0.66 +/- 0.024	0.75 +/- 0.022	0,7673	
874.7	di21:0 PC	Standard			
468.5	14:0 LPC	0.04 +/- 0	0.05 +/- 0.004	0.620	
494.5	16:1 LPC	0.12 +/- 0.013	0.14 +/- 0.004	0.388	
496.5	16:0 LPC	0.43 +/- 0.031	0.53 +/- 0.020	0.0006	***
520.5	18:2 LPC	0.11 +/ 0.009	0.16 +/- 0.011	0.043	*
522.5	18:1 LPC	0.84 +/- 0.058	0.79 +/- 0.031	0.072	
524.5	18:0 LPC	0.087 +/- 0.009	0.16 +/- 0.009	0.008	**
538.5	19:0 LPC	Standard			

PC and LPC molecular species were assessed in SW620 and HT29 cells by positive mode ESI-MS/MS as described in Methods. Precursor ions m/z are shown with the corresponding PC/LPC species. Data are expressed as mean (pmoles/µg proteins) ± s.e.m. *P*-values were determined by multiple student's t-test. *p<0.05. **p<0.01. ***p<0.001.

Supplementary Table 2. Patient characteristics (n=79)

**Age (years)**	Mean 63 range [29-83]
**Sexe**	
Males	55
Females	24
**Tumor origin**	
Colon	50
Rectum	29
**TNM stage**	
T stage	
1-2	0
3-4	50
N stage	
0	16
1-2	34
M stage	
0	21
1	29
missing	29
**Microsatellite status**	
Stable	79
Instable	0
**Ras status**	
WT	17
Mutated	11
missing	51
**Braf status**	
WT	23
Mutated	1
missing	55
**Neoadjuvant treatment**	
Yes	54
No	25

Supplementary Table 3. Primers used for RT-qPCR analysis

Gene	Forward sequence	Reverse sequence
h-*Lpcat2*	5’-TTGTTGCTGCCCCTCATTCA-3’	5’-GGGAATCCGGATCTACACGG-3’
h-*Plin2*	5’-CATTGTGTTGCCTCTGTTAC-3’	5’-CCTTTTCAGATCACACCAGA-3’
h-*Lpcat1*	5’-CCTCATGACACTGACGCTCTTC-3’	5’-CAGGAAGTCCACAACCTTCCTC-3’
h-*Pyct1A*	5’-ATGCACAGTGTTCAGCCAA-3’	5’-GGGCTTACTAAAGTCAACTTCAA-3’
h-*Lpcat3*	5’-ATACTATTACACTGCCACCGGCAA-3’	5’-AGTAGGAGAAACCAGCAACTTCCA-3’
h-*Lpcat4*	5’-CTGCAGCATTGTGGATGTGG-3’	5’-TGTGCCATGACCCTCTGAAC-3’
h-*Chpt1*	5’-ATTGTTGCTCCTCCAAATGC-3’	5’-GCGCTCATTGGCAGACTTAT-3’
h-*Epas1*	5’-TACAATCCTCGGCAGTGTCCT-3’	5’-ACGGAGAGAAGGGAACCCAA-3’
h-*Plin1*	5’-GACCTCCCTGAGCAGGAGAAT-3’	5’-GTGGGCTTCCTTAGTGCTGG-3’
h-*Plin3*	5’-CAGGAACAGAGCTACTTCG-3’	5’-CAGTTTCCATCAGGCTTAGG-3’
h-*Plin4*	5’-CCTGGACCACTCCCTTAG-3’	5’-CAACCTTCGGAAAAGCAGAT-3’
h-*Plin5*	5’-TTTTGGTATTGGACTCTCCCATTT-3’	5’-CTCCCCCGGGCCTCTT-3’
h-*ChkA*	5’-CACTTTCCGAAGCTCATCAC -3’	5’-GGACGAGTTCCACATCAGTGT -3’
h-*Actb*	5’-AGAGCTACGAGCTGCCTGAC-3’	5’-AGCACTGTGTTGGCGTACAG-3’
m-*Lpcat2*	5’-GCTCCTTTCATCTCACATTG-3’	5’-CGGTGTTGATTAATTGCTGA-3’
m-*Actb*	5’-ATGGAGGGGAATACAGCCC-3’	5’-TTCTTTGCAGCTCCTTCGTT-3’

Supplementary Table 4. Antibodies

Antibody against:	Reference #	Providers	Application
GAPDH	G9545	Sigma Aldrich	Western-blotting (WB)
PLIN2. clone ADFP-5	SAB4200452	Sigma Aldrich	
β-Actin. clone AC-74	A2228	Sigma Aldrich	
CHKA	HPA024153	Sigma Aldrich	
LPCAT1	HPA022268	Sigma Aldrich	
LPCAT4	HPA030719	Sigma Aldrich	
m-LPCAT2	SAB4502278	Sigma Aldrich	
PLIN3	HPA006427	Sigma Aldrich	
Caspase 12	ab62484	Abcam	
CHPT1	ab107425	Abcam	
Calreticulin. clone D3E6	12238	Cell Signalling	
Caspase 3. clone 8G10	9665	Cell Signalling	
Caspase 8. clone 1C12	9746	Cell Signalling	
Caspase 9	9502	Cell Signalling	
PARP	9542	Cell Signalling	
FADD	2782	Cell Signalling	
Phospho-eif2α. clone D9G8	3398	Cell Signalling	
total eif2α. clone D7D3	5324	Cell Signalling	
Bip. clone C50B12	3177	Cell Signalling	
CHOP. clone D46F1	5554	Cell Signalling	
CCTα. clone D18B6	6931	Cell Signalling	
HIF2α. Clone D9E3	7096	Cell Signalling	
PLIN1	9349	Cell Signalling	
HSC-70. clone B-6	sc-7298	Santa Cruz Biotechnology	
Calnexin. clone H-70	sc-11397	Santa Cruz Biotechnology	
PLIN4	PA5-60792	ThermoFisher scientific	
PLIN5	PA1-46215	ThermoFisher scientific	
h-LPCAT2	HPA007891	Sigma Aldrich	WB/IF(microscopy)/IHC
CD8. clone C8/144B	M7103	Dako	IHC
Calreticulin. clone FMC75	ab22683	Abcam	IF (microscopy and cytometry)
PDI	2446	Cell Signalling	IF
PLIN2. clone EPR [3713]	ab108323	Abcam	Cytometry
CD45-APC-Cy7. clone C30-F11	557659	BD Biosciences	
CD3-FITC. clone 145-2C11	553061	BD Biosciences	
CD8-BV605. clone 53-6.7	563152	BD Biosciences	
PD-1-PE. clone J43	551892	BD Biosciences	
Ki67. Clone B56	550609	BD Biosciences	
Tim-3-APC. clone REA-602	130-109-448	Miltenyi Biotech	
